# Supplementary material for: Annotating digital text with phonemic cues to support decoding in struggling readers
Source: PLoS One. 2020 Dec 7;15(12):e0243435. doi: 10.1371/journal.pone.0243435 (PMC7721157; doi:10.1371/journal.pone.0243435)
Supplement: S1 Table — Listed are the responses to the post-study questionnaire for the intervention group participants and their parent. After completing the study, children were asked to answer honestly to the following questions: Did you like the app? And would you like to use the app again in the future? Parents were then asked if they enjoyed using the app. Those adults who did not respond did not participate in the practice to comment on the app. (DOCX) [file pone.0243435.s001.docx]

S1 Table. Parent/child responses to post study questionnaire for the intervention group

|  | **Yes** | **No** | **Maybe** | **Did not respond** | **Total** |
| --- | --- | --- | --- | --- | --- |
| **[Child] "Did you like the app?"** | 17 | 2 | 0 | 1 | 20 |
| **[Child] "Would you use the app again in the future?"** | 17 | 1 | 1 | 1 | 20 |
| **[Adult] "Did you like the app?"** | 16 | 0 | 0 | 4 | 20 |

Listed are the responses to the post-study questionnaire for the intervention group participants and their parent. After completing the study, children were asked to answer honestly to the following questions: Did you like the app? And would you like to use the app again in the future? Parents were then asked if they enjoyed using the app. Those adults who did not respond did not participate in the practice to comment on the app.
