# Supplementary material for: Annotating digital text with phonemic cues to support decoding in struggling readers
Source: PLoS One. 2020 Dec 7;15(12):e0243435. doi: 10.1371/journal.pone.0243435 (PMC7721157; doi:10.1371/journal.pone.0243435)
Supplement: S2 Table — Frequency information for real word stimuli. Words were retrieved from MCWord Orthographic Wordform Database (http://www.neuro.mcw.edu/mcword/). According to the database, the frequency is a measure of how often the wordform occurred in 1,000,000 presentations in the CELEX database. (DOCX) [file pone.0243435.s002.docx]

S2 Table. Real word frequency statistics

| **Word List 1** | |  | **Word List 2** | |  | **Word List 3** | |  | **Word List 4** | |
| --- | --- | --- | --- | --- | --- | --- | --- | --- | --- | --- |
| **Word** | **Frequency** |  | **Word** | **Frequency** |  | **Word** | **Frequency** |  | **Word** | **Frequency** |
| has | 1809.24 |  | his | 5828.33 |  | had | 6491.25 |  | can | 1954.34 |
| you | 0.18 |  | now | 1683.88 |  | she | 4270.81 |  | one | 3302.38 |
| was | 11050.2 |  | who | 4754.07 |  | the | 61445.7 |  | for | 8319.47 |
| not | 5082.53 |  | are | 4254.92 |  | all | 3510.01 |  | her | 4032.24 |
| but | 5240.18 |  | him | 2617.38 |  | and | 28470.7 |  | out | 2521.24 |
| then | 1820 |  | were | 3589.55 |  | this | 4504.73 |  | only | 1774.31 |
| from | 4229.52 |  | time | 1770.21 |  | when | 2560.63 |  | into | 2006.57 |
| they | 4734.61 |  | what | 2581.57 |  | that | 11437.3 |  | have | 4529.54 |
| been | 2716.62 |  | more | 2427.66 |  | will | 2105.87 |  | them | 2305.28 |
| said | 2838.7 |  | like | 1884.02 |  | with | 7066.31 |  | some | 1859.62 |
| again | 766.802 |  | other | 1596.96 |  | about | 2322.72 |  | right | 758.533 |
| could | 1906.39 |  | world | 739.197 |  | where | 1034.58 |  | those | 866.215 |
| their | 2947.33 |  | after | 1185.51 |  | which | 3330.76 |  | would | 2913.48 |
| first | 1210.08 |  | still | 923.923 |  | years | 899.53 |  | never | 906.253 |
| these | 1223.29 |  | being | 850.746 |  | there | 3232.06 |  | think | 800.297 |
| called | 416.152 |  | course | 604.387 |  | always | 651.267 |  | social | 408.299 |
| though | 639.428 |  | during | 390.391 |  | enough | 500.87 |  | mother | 446.196 |
| little | 972.588 |  | around | 514.136 |  | people | 1317.05 |  | should | 956.941 |
| looked | 531.865 |  | really | 484.271 |  | rather | 471.718 |  | almost | 481.177 |
| things | 535.018 |  | seemed | 437.272 |  | before | 987.282 |  | better | 435.784 |
| looking | 292.942 |  | without | 595.76 |  | another | 690.83 |  | certain | 279.437 |
| against | 600.996 |  | society | 270.752 |  | however | 435.546 |  | whether | 308.41 |
| between | 742.529 |  | perhaps | 444.292 |  | because | 1166.95 |  | thought | 707.012 |
| nothing | 520.74 |  | already | 373.02 |  | country | 335.123 |  | british | 353.327 |
| himself | 529.664 |  | morning | 305.257 |  | through | 956.346 |  | general | 300.081 |
| happened | 187.997 |  | american | 248.977 |  | although | 304.841 |  | students | 178.002 |
| probably | 263.731 |  | thinking | 173.124 |  | business | 237.733 |  | position | 194.601 |
| together | 364.691 |  | remember | 238.447 |  | children | 647.817 |  | possible | 345.593 |
| question | 238.685 |  | problems | 221.194 |  | economic | 197.337 |  | interest | 211.437 |
| national | 240.351 |  | suddenly | 192.757 |  | anything | 380.575 |  | movement | 192.697 |
|  |  |  |  |  |  |  |  |  |  |  |
| **MEAN:** | 1821.7683 |  | **MEAN:** | 1406.065433 |  | **MEAN:** | 5065.474833 |  | **MEAN:** | 1488.292067 |

Frequency information for real word stimuli. Words were retrieved from MCWord Orthographic Wordform Database (<http://www.neuro.mcw.edu/mcword/>). According to the database, the frequency is a measure of how often the wordform occurred in 1,000,000 presentations in the CELEX database.
