# Supplementary material for: Annotating digital text with phonemic cues to support decoding in struggling readers
Source: PLoS One. 2020 Dec 7;15(12):e0243435. doi: 10.1371/journal.pone.0243435 (PMC7721157; doi:10.1371/journal.pone.0243435)
Supplement: S3 Table — Frequency information for pseudoword stimuli. Pseudowords were retrieved from MCWord Orthographic Wordform Database (http://www.neuro.mcw.edu/mcword/). According to the database, the constrained bigram frequency is a measure of how often the bigram wordform occurred in 1,000,000 presentations in the CELEX database. (DOCX) [file pone.0243435.s003.docx]

S3 Table. Pseudoword frequency statistics

| **Pseudo List 1** | |  | **Pseudo List 2** | |  | **Pseudo List 3** | |  | **Pseudo List 4** | |
| --- | --- | --- | --- | --- | --- | --- | --- | --- | --- | --- |
| **Word** | **Constrained Bigram Frequency** |  | **Word** | **Constrained Bigram Frequency** |  | **Word** | **Constrained Bigram Frequency** |  | **Word** | **Constrained Bigram Frequency** |
| cru | 26.27 |  | hov | 641.66 |  | ope | 3 |  | lan | 1792.94 |
| ers | 199.98 |  | hal | 4326.94 |  | sed | 1185.45 |  | hel | 2032.99 |
| oth | 0.09 |  | med | 730.3 |  | ery | 220.18 |  | hin | 4364.75 |
| nup | 38.46 |  | ute | 21.42 |  | urt | 83.74 |  | nef | 558.55 |
| por | 4284.4 |  | ent | 231.52 |  | wim | 1435.05 |  | irt | 94.5 |
| mish | 818.92 |  | deen | 5215.02 |  | fass | 1629.96 |  | tast | 3249.51 |
| thub | 9045.93 |  | clim | 99.06 |  | fust | 2993.5 |  | pind | 1746.77 |
| flad | 764.48 |  | pess | 894.12 |  | cark | 1815.14 |  | dard | 1288.16 |
| feen | 5399.92 |  | wilm | 4372.96 |  | wilk | 4509.2 |  | felp | 1568.29 |
| trag | 262.78 |  | fack | 2171.32 |  | surn | 897.63 |  | thip | 10553.53 |
| bress | 1356.15 |  | yound | 3047.7 |  | grong | 2267.25 |  | keeds | 695.47 |
| stath | 1763.17 |  | sweel | 538.04 |  | doint | 2120.59 |  | prent | 1132.06 |
| parge | 1075.33 |  | lorks | 1110.74 |  | wouth | 3990.52 |  | terve | 535.82 |
| tound | 3095.77 |  | frugs | 361.54 |  | twisp | 192.3 |  | blace | 1631.47 |
| draff | 593.23 |  | thich | 6451.07 |  | greep | 1211.39 |  | dring | 2763.72 |
| calent | 1050.81 |  | plampy | 320.15 |  | balket | 1074.27 |  | prined | 2646.82 |
| empand | 560.47 |  | twelds | 283.03 |  | freety | 536.92 |  | strust | 846.43 |
| skults | 328.28 |  | moddle | 1412.67 |  | strope | 813.88 |  | hormal | 872.82 |
| befort | 1546.42 |  | rember | 2182.66 |  | peemed | 2620.72 |  | garked | 2695.43 |
| natual | 939.25 |  | insing | 2129.97 |  | degair | 411.63 |  | danted | 3001.98 |
| finands | 672.77 |  | harning | 3659.92 |  | cappors | 934.04 |  | subbery | 920.38 |
| himsent | 968.93 |  | plastly | 792.64 |  | musteme | 631.52 |  | gettled | 2100.96 |
| traffit | 319.36 |  | spanged | 1770.06 |  | bastily | 1244.5 |  | kithods | 744.23 |
| propind | 2381.8 |  | sibbing | 3107.28 |  | tobbing | 3086.9 |  | grovice | 838.9 |
| putsins | 2074.72 |  | ordular | 237.39 |  | groblex | 788.46 |  | pervice | 888.55 |
| contrage | 686.77 |  | crinking | 1868.92 |  | chamater | 860.61 |  | swincher | 642.34 |
| identant | 435.02 |  | natching | 1896.4 |  | marasand | 455.46 |  | dwending | 1719.41 |
| probling | 2309.61 |  | offector | 298.51 |  | amervise | 372.2 |  | inforway | 380.47 |
| retraver | 933.99 |  | pastrows | 301.7 |  | haparder | 653.43 |  | felegral | 446.09 |
| preaming | 2153.14 |  | interter | 1186.8 |  | feetched | 1285.6 |  | salcatic | 263.92 |
|  |  |  |  |  |  |  |  |  |  |  |
| **MEAN:** | 1536.207333 |  | **MEAN:** | 1722.050333 |  | **MEAN:** | 1344.168 |  | **MEAN:** | 1767.242 |

Frequency information for pseudoword stimuli. Pseudowords were retrieved from MCWord Orthographic Wordform Database (<http://www.neuro.mcw.edu/mcword/>). According to the database, the constrained bigram frequency is a measure of how often the bigram wordform occurred in 1,000,000 presentations in the CELEX database.
