# Supplementary material for: Annotating digital text with phonemic cues to support decoding in struggling readers
Source: PLoS One. 2020 Dec 7;15(12):e0243435. doi: 10.1371/journal.pone.0243435 (PMC7721157; doi:10.1371/journal.pone.0243435)
Supplement: S1 File — Each passage is provided as well as the comprehensions that were used to determine passage completion. Additional analyses, with explanations, are also provided. (PDF) [file pone.0243435.s004.pdf]

S1 File. At-home practice passages and comprehension questions.

Contents

Supplementary Analyses 2
At-Home Practice Passages 3
Second Grade Passages 3
Third Grade Passages 9
Fourth Grade Passages 16
Comprehension Questions 22
Second Grade Passages 22
Third Grade Passages 23
Fourth Grade Passages 24

Supplementary Analyses

Role of Age and Phonological Awareness as Covariates to Mixed Effects Models

Due to the heterogeneity of our struggling reader population in both reading ability and age, we performed an exploratory analysis of these results testing a model that adds covariates for participant age and initial phonological awareness ability (as measured using the CTOPP phonological awareness composite measure).

Model fits were compared using AIC/BIC values and revealed that in no case was the model with added covariates a superior model to the simpler model used in the manuscript. Looking at the results of these models, moreover, revealed no significant effects of age or phonological awareness. Interaction effects were unchanged from the simpler models reported in the manuscript.

The model fit workflow and code associated with this analysis can be found in the associated project repository on GitHub.

At-Home Practice Passages

Second Grade Passages

Lily's Time Line

Lily wanted to do a project. "What about making a time line?" her father asked. "That is a list of important events in your life."

"Sure!" Lily replied. "Can you show me how?"

Lily's dad gave her a piece of paper, a pencil, and a ruler. She held the pencil and used the ruler to draw a straight line. Then her father marked a small line across the longer line at the left end. Above the small line, he wrote, Lily is born.

"Why did you make that small line at the left end, Dad?" Lily asked.

"On a time line, events are ordered from left to right. The first event is placed on the left end and the last event is placed on the right end."

Lily then wrote the month, day, and year of her birthday under the first small line.

Next, her dad marked another small line to the right of the first small line. He wrote, Lily learns to walk above that line. Then he marked another small line to the right of the second small line. Above this line he wrote, Lily starts school.

"What about when I learned to ride my bike?" asked Lily.

"Right! We should add that," her dad said.

"I want to add pictures," Lily told her dad. She made a drawing of herself riding a bike and taped it to the paper. Her dad put a photo of Lily's first day of school on the time line, too.

Now their work was done. Lily said, "That time line is a picture of my life." "It is a picture of your life so far," her dad added. "There is a lot more to come!"

At the Lake

Layla and her family were driving to the town park. It was a hot day. She wanted to swim in the lake. As they got out of the car, Layla saw her friends.

"Let's go into the water!" she called.

Layla always had fun at the park in the summer. Families had picnics. The kids played and went swimming in the lake.

In the spring, Layla's dad took her fishing at the lake. They went early in the morning. The lake water was still. It was different from the river, which had water that rushed by.

One morning in the fall, the lake had fog over it. Layla's dad told her how fog formed. He said hot summer days had warmed the water. Then cooler fall air moved over the lake's warm water. The water vapor, or the gas form of water, in the air became cool. That caused condensation. It changed the gas into tiny drops of water. We can see those drops of water hang in the air as fog.

Layla loved winter at the park, too. The cold temperatures made the lake freeze. When the ice was thick and safe, people went ice-skating.

The lake changed from season to season. But it was always a place to have fun!

SummerReads: Moths – Silkworms

Silkworms are the caterpillars of the silk moth. The eggs of the silk moth are tiny and the caterpillars that come out when the eggs hatch are tiny. But these silkworms do not stay tiny for very long. They eat and grow until the caterpillar is about the length of your longest finger. At this point, the caterpillars build cocoons.

Over two to three days, the caterpillar spins about one mile of thread until it is completely covered in its cocoon. This cocoon is special since it is made from a single long thread that is very strong.

The thread of the cocoon is used to make silk for things like clothes and rugs. Other moth caterpillars also make silk threads. But the silkworm makes the longest and best silk thread. Even so, it takes a lot of silk thread to make something people can use or wear. For example, it takes about 150 cocoons to make just one silk tie that a man might wear.

Silkworms were once found in the wild of Asia. But for thousands of years, silkworms have been kept in farms that are mostly in Asia. At these farms, silkworms are kept on trays covered with special paper.

You won't see any silkworms outside this summer. But you will probably see another kind of animal that also makes silky threads—spiders!

The Volcano That Keeps Erupting

A volcano on the island of Hawaii has been erupting since 1983. The volcano's name is Kilauea (kee-lah-WAY-ah). It has released tons of hot, melted rock called lava.

Volcanoes are openings on the surface of our planet that can send lava, gas, and steam into the air. You may have seen volcanoes that look like mountains with openings at the top. Many volcanoes look like that. But others appear more flat. An example of a flatter volcano is the shield volcano.

Most of the world's volcanoes are found in an area that forms a shape like a horseshoe around the Pacific Ocean. Scientists call that area the "Ring of Fire."

Kilauea is a shield volcano. Lava from Kilauea has done a lot of damage. In 1990, lava flowed over a hundred homes, a church, and a store in the village of Kalapana. Those places were destroyed. From 1983 to 2011, lava destroyed almost all the houses in another community called Royal Gardens. There was one house that survived all those years. In 2012, another lava flow ruined the only home remaining in that community. Today, Royal Gardens is no longer home to anyone.

Scientists say Kilauea's lava threatens more homes and a forest preserve. The scientists are using computers to map the lava's path. They hope to predict what Kilauea will do in the future.

The Time Machine

"So you're telling me this is a time machine?" Eric asked. "Yes," his Uncle Joseph replied. "I've been working on it for years here in my lab." Eric looked around the lab. A bank of computers lined one of the walls. Metal shelves held bottles and jars filled with strange liquids. There was a big metal table in the middle of the room. There were wires and tiny machine parts scattered all over it. In the middle of the table was a silver box. The box had a small computer screen and keyboard on its face.

"All you do is type in the time you want to go to," Uncle Joseph said. "The box will transport anyone within ten feet to that location." "So you mean if you typed in yesterday's date, it would send us back to yesterday?" Eric asked. Uncle Joseph nodded. "Yes. But it can do better than that. I could type in 150 million years BC, for example."

He typed in the date on the keyboard. "Cool," Eric said. "And then you just press this button?" Eric put his finger on the button. "Eric, no!" Uncle Joseph cried. But he was too late. Eric pressed the button. The room began to spin. When everything settled, they were no longer in the lab. Tall, green plants grew all around them. A volcano rose up in the distance. Then the ground began to rumble. A huge dinosaur stomped toward them! "Hey, a Brontosaurus!" Eric said. "Your time machine really works!"

A Clean Park

Tyler ran into the kitchen. "Mom, I'm going to the park!" he said."Wear your helmet!" his mom called back. The park was just down the street. Tyler grabbed his bike and rode there. It was pretty much his favorite place to go. The park looked pretty crowded. Some kids were playing. But other kids held garbage bags. They were picking up trash.

Tyler stopped his bike. One of the kids was Lauren, a girl from his school. She waved at Tyler. "We're cleaning up the park today," she said. "Want to help?" "No thanks," Tyler said. Today was Saturday, a day off. He came to the park to have fun, not to work!

Tyler rode his bike around the path. He passed the swings and slide. He passed the small pond. As he rode, he saw things he never noticed before. There was lots of garbage on the ground. He saw old soda bottles. He saw old food wrappers. It was pretty gross.

Tyler rode back to Lauren. He got off his bike. “I’ll help,” he said. Lauren smiled. “Here,” she said. She gave him a bag. Tyler and the kids cleaned for about an hour. When they were done, the park looked great. Tyler rode his bike around the path again. There were no old soda bottles. There were no old food wrappers. Tyler smiled. Now the park was better than ever!

Martin’s Birdhouse

Drawing a picture can be fun. Drawing can also help people communicate. Putting an idea on paper is sometimes a good way to solve a problem.

Here is an example. Martin wanted to build a new birdhouse. The last one he had built fell apart after one windy night. This time, Martin knew he had to make a stronger birdhouse. He just was not sure how to do that.

Martin asked his friend Diego for help. Diego was good at building things. Martin explained his idea about the birdhouse, but Diego was confused. So Martin drew a picture to show what he was thinking.

Diego looked at the sketch carefully. “Why does the hole for the bird need to be so big?” he asked Martin. “It doesn’t,” said Martin. “You’re right.” Diego also suggested that the birdhouse could be attached to the tree in a much better way. He drew another picture to show Martin how that attachment would work.

After talking a while longer, Martin and Diego drew one more sketch. Then they began building the birdhouse together. “We are a good team,” Martin told Diego. “I think your ideas will make my birdhouse stronger!”

I Want to Buy a Computer Game

Luis was excited. The new computer game he wanted was finally in stores. “The game is here!” he told his mom. “Can we go buy it?”

“How much does it cost?” Luis’s mother asked.

“Thirty-five dollars,” he replied.

“That is a lot of money, Luis. Do you have enough to buy it yourself?”

He shook his head.

“You know, Luis, we have to spend our money carefully. We have to pay for our house and food. We need to buy clothing and books and gas for our car. Our money goes to things we need.”

“But I really want this game!” answered Luis. “What can I do?”

“You get eight dollars a week for doing chores,” his mom said. “Try to save it. Before long, you will have enough to pay for the game.”

“I do not think so,” said Luis. “By then, all the games will be sold.”

“Try it,” replied his mother.

Weeks later, Luis came home very happy. “Guess what, Mama? I did what you said. I saved my chore money. Then I saw the game was on sale. Today I bought it for twenty-eight dollars.”

“And,” his mother added, “you did it with your own money!”

Oh Nuts!

Chestnut and Daisy were two young squirrels. They were born in the same spring month. They had been best friends ever since.

The two friends had fun all summer. They raced each other along the top of the long fence around the park. They climbed to the tops of the tallest trees. They took naps under the raspberry bush. They ate delicious scraps of food that people left behind in the park.

Then fall came. Chestnut didn’t want to play anymore. He spent every day collecting the nuts that fell from the trees. Then he buried them all over the park. “Nuts, nuts, nuts,” said Daisy. “Can’t you think about anything else?”

“But winter is coming soon,” Chestnut warned her. “We’ll need something to eat then.”

“We can eat scraps that people leave behind, like we always do,” Daisy said. “Maybe we can,” Chestnut said. “But I’d rather be safe than sorry.”

So Chestnut kept collecting nuts. But Daisy ran along the top of the fence by herself. She climbed the tops of the tallest trees. She took lots of naps.

Then winter came. People stopped coming to the park. There were no scraps of food to eat. Daisy became very hungry. But Chestnut had plenty of nuts to eat. “Chestnut, can I please have some of your nuts?” Daisy asked.

“Of course you can,” Chestnut said. “Just promise you’ll help me collect nuts next fall.”

“I will!” Daisy promised. “You were right all along!”

The Rocky Mountains

The Rocky Mountains are a large group of mountains on the North American continent. The Rockies are found in the western region of North America. They stretch from Canada in the north to the United States in the south. The southern part of the Rockies ends in the state of New Mexico. The Rocky Mountains also go through the states of Montana, Idaho, Wyoming, and Colorado.

The Rockies are the longest mountain range in North America. They have many high peaks. The highest is Mount Elbert in Colorado. It is nearly three miles high. Mount Elbert’s peak is fourteen thousand feet above sea level.

In the summer, the Rockies are warm and dry, though it does rain sometimes. During the winter, the weather is wet and very cold. Heavy winter snow falls in the higher parts of the Rockies.

Many people like to visit these mountains. Here they ride bikes, hike on trails, ski, or fish. A special area called Rocky Mountain National Park is in Colorado. The national park is reserved for people to visit. Visitors to Rocky Mountain National Park are excited to see the wild animals that live there. Those animals include elk, moose, mountain goats, and mountain sheep.

A Time of Dust Storms

A long time ago, wind and dust caused big problems in the United States. Giant clouds of dust harmed people, animals, and crops in the central part of the country. The area became known as the Dust Bowl.

The problem started when farmers planted more crops than they could sell. The next year, those farmers decided to leave some land empty. The farmers let their cattle graze there, and the cattle ate the grass. Soon, nothing grew on that land.

Then came a drought (DROWT*)*. A drought is a long period without rain. Land became very dry. Grass and crops barely grew. Very little was left to hold the soil in place.

At the same time, strong winds started blowing. The winds blew layers of soil into the air. Dust covered everything.

People had to protect themselves from the dust. Children wore masks and goggles when they walked to school. They used the masks so they would not breathe in dust and get sick. People hung wet sheets over their windows to prevent dust from blowing into their homes.

Finally, the rains returned. Farmers could plant crops again. The farmers learned to plant in different places to protect the soil. They also planted grasses to keep the soil in place.

When Television Became Colorful

Today, nearly all television programs are broadcast in color. If you turn on a baseball game, you can see that the grass on the field is green, or that the pitcher has a blue cap on. But when your grandparents were children, most people watching TV at home could not have seen any of those colors. Television programs were broadcast in black and white only.

Television sets that could broadcast in color have been around for a long time. An engineer named John Logie Baird invented a color TV set in the 1930s. But the picture on Baird's TV flickered, and was not clear. Companies would not sell a TV that was not good quality.

For many years, people worked to improve how color televisions worked. Over time, companies found ways to make the picture clearer. The improvements also meant that a user could turn a dial to add just the right amount of color to the picture.

By the late 1960s, many people were buying color televisions. Soon, most TV shows were being broadcast in color, and most people in the U.S. had color TV sets.

Today, it’s unusual to find any television show that is still broadcast in black and white. Now the world of television is full of color!

Third Grade Passages

Return of the Bear

Grizzly bears are huge, powerful animals. Thousands once lived in the western United States. In recent years, however, the number of bears has been very low.

Over time, illegal hunting and habitat loss caused many bears to die off.

A habitat is a place in nature where an animal lives. The bears' habitat became smaller when people cut down trees to build homes and roads.

Many people thought they would never again see a grizzly in the West. Now wildlife officials say the bears are making a comeback.

Good News for Grizzlies

Grizzly bears live in Yellowstone National Park and the surrounding forests. That area covers parts of the western states of Wyoming, Montana, and Idaho. Today, more than 550 grizzlies live there. That is almost double the number that lived there in the 1980s!

The bears living in the Yellowstone area have been protected by the Endangered Species Act. Wildlife officials say these bears may be taken off the endangered species list within the next year.

"That does not mean the bears will no longer be protected," grizzly bear expert Chris Servheen told *Weekly Reader*. "We will continue to monitor them to make sure they are healthy. A lot of people are interested in making sure grizzlies survive."

What is Light?

Did you know that the sun is the greatest source of light for our planet, Earth? But what is light? Why is it so important?

Hot gases of the sun give off both light and heat energy. Light carries energy, with the long wavelengths carrying the least and the short wavelengths carrying the most. When you think of something with lots of energy, what comes to mind? Do you think of something fast like a race car?

Do you think of something with great force like a very strong wind knocking down a tree?

Believe it or not, light can be many times more energetic than a car or the wind.

Light travels at 186,000 miles every second in a vacuum. At that speed, light can go around Earth more than seven times every second! No human-made machine can go that fast—not even a jet plane or rocket!

One way that light travels, including light from the sun, is in the form of waves. Scientists can measure how long light waves are. Waves can be different sizes—some are long and some are short. Some light waves are visible and some are invisible. Whether you can see light or not depends on the length of the wave. The longest wavelength of visible light is seen as red and the shortest wavelength is violet. Short wavelengths carry the most energy.

SummerReads: Lakes – The Great Salt Lake

The water from most lakes tastes like the water from a tap in your house. This kind of water is called freshwater. Almost all lakes are filled with freshwater. However, the water in the Great Salt Lake of Utah is saltwater, not freshwater. In fact, it is up to nine times saltier than the saltwater in the oceans. If you tried to drink this water, you would want to spit it as fast as you could.

The water that flows into lakes from rivers first falls to earth as rain or snow. Rainwater or snow tastes fresh, not salty. The water that runs into the Great Salt Lake, like all lakes, is fresh. What happens to the water once it runs into the Great Salt Lake to make it so salty?

As the water runs over the ground, it touches rocks and soil. Some parts of the rocks and soil break up or dissolve in water. As this water flows into the Great Salt Lake, it brings in dissolved materials, such as salt. Unlike most lakes, the Great Salt Lake does not have streams and rivers that flow out of it. The dissolved material, including salt, cannot flow out of the lake. Water can only leave the Great Salt Lake through evaporation. Evaporation is when water turns into a gas in the air. Evaporation only takes pure water out of the lake. The salt does not evaporate. It stays in the lake. That’s why there is a very salty lake in Utah, far from any ocean.

Trouble in the Amazon

Why is this rain forest shrinking so quickly?

Bright-colored birds fly through the air. Monkeys leap from tree to tree. Jaguars creep on the ground below. Those are just a few of the thousands of animals that live in the Amazon rain forest.

The Amazon in South America is the largest tropical rain forest in the world. A tropical rain forest is a thick forest in a warm region with heavy rainfall.

People are destroying the Amazon. Each year, farmers and loggers cut down rain forest trees to make room for farms, homes, and roads. Scientists have recently discovered that the Amazon is shrinking twice as quickly as they once thought.

Cause for Concern

The Amazon rain forest is one of the richest areas of the world in animal and plant diversity, or variety. Many of the foods, spices, and medicines people need come from the Amazon.

The Amazon rain forest also helps recycle Earth’s air. The trees give off oxygen for people to breathe. Trees also clean the air by taking in carbon dioxide. Too much carbon dioxide in the air is harmful to humans.

Fixing the Problem

Stopping people from destroying the rain forest is not an easy task. "Almost 2 million people make the forest their home," scientist Jim Bowyer told *Weekly Reader.* "All these people need land for farming and wood for heat and cooking. They are looking for a way to survive. Solutions need to involve the very people who destroy the forest."

Did You Know?

Rain forest trees stay green all year long.

The tallest trees may grow up to 200 feet!

When leaves and branches fall, they break down and release nutrients into the soil.

Australia’s Natural Wonder: The Great Barrier Reef

In the Coral Sea in Australia, there is a reef. A reef is a ridge of rock, coral, or sand near the surface of a sea. But this reef isn’t just any old reef. It’s the Great Barrier Reef, the world’s largest coral reef system. It covers over 2300 kilometers, reaching from shallow areas to deep ocean waters. It is so big that it can be seen from outer space!

The Great Barrier Reef is home to many types of living things. It has thousands of types of mollusks and over 1500 kinds of fish. It’s also home to many species of sharks and dolphins, not to mention sea turtles, sea cows, and other creatures. Humpback whales even find their way to the reef to breed!

Although it’s home to many creatures, the reef might be best known for its coral. The Great Barrier Reef has over 400 different kinds of coral. It includes both soft and hard coral. The reef’s corals are many different colors, shape, and sizes. This is partly what makes the reef so beautiful.

The Great Barrier Reef is known as one of the most diverse and beautiful places in the world. But today, warmer ocean temperatures are putting great stress on the coral. Pollution also affects the reef, as sediments or litter often find their way into the water. This is bad for the health of the reef and its plants and animals. People are working hard to protect the Great Barrier Reef and the species that live there.

The Northern Lights

Imagine you are somewhere far north on Earth. It's nighttime, but the sky is not dark, like you might expect it to be. Instead, it's filled with colorful lights. Some lights look like moving curtains. Others look like a steady glow, or bands across the sky. The lights are usually green, but you may see other colors, like yellow, red, purple, or blue. The lights are dazzling as they dance far above your head.

What you're imagining is the Aurora Borealis. It's also called the Northern Lights. This natural light display has wowed people for hundreds of years. In fact, it is one of the seven natural wonders of the world.

People can thank the sun for these incredible light shows. During a certain kind of solar storm, energy and small particles from the sun can travel all the way to Earth. They can enter the atmosphere near the North Pole. These particles interact with the gases in our atmosphere. This causes the beautiful lights we see in the sky.

The color of the lights partly depends on the gas that interacts with the particles from the sun. Oxygen gives off green light or red light. Purples and blues come from nitrogen in the atmosphere.

The same kind of light show can happen near the South Pole, as well. That display is called the Aurora Australis.

It's usually easiest to see the Northern Lights in a place where it is dark. It also helps to be far north on Earth, in places such as Canada, Iceland, Alaska, and Greenland. And, of course, you're most likely to see the lights after there's a big solar storm!

Sunflowers

A sunflower is a big, circular, yellow flower. Sunflowers need a lot of sun to grow. Sunflowers are actually made up of lots and lots of tiny flowers. The center part is made of one kind of flower, and the petals around it are another kind of flower.

We use sunflowers in different ways. One thing we do with them is look at them! Many people add them to gardens because they are so big, bright, and colorful. They can also be cut and brought inside. They will last a long time in a vase. A vase is a jar, bottle, or other container that is used to hold flowers.

Sunflower seeds are good to eat. People, birds, and other animals, including squirrels and chipmunks, love to eat sunflower seeds. They can be difficult to eat if they are still in their shells, but they are filled with protein and are good for you! Sunflower seeds also have a lot of oil in them. It can be squeezed out and collected. Many people use sunflower oil for cooking.

Sunflowers are pretty flowers, and they give us and other animals food. Be careful of the stems, though—they are rough and very scratchy!

The Fox and the Crow
From "Aesop's Fables"

One bright morning as the Fox was following his sharp nose through the wood in search of a bite to eat, he saw a Crow on the limb of a tree overhead. This was by no means the first Crow the Fox had ever seen. What caught his attention this time and made him stop for a second look, was that the lucky Crow held a bit of cheese in her beak.

"No need to search any farther," thought sly Master Fox. "Here is a dainty bite for my breakfast."

Up he trotted to the foot of the tree in which the Crow was sitting, and looking up admiringly, he cried, "Good-morning, beautiful creature!"

The Crow, her head cocked on one side, watched the Fox suspiciously. But she kept her beak tightly closed on the cheese and did not return his greeting.

"What a charming creature she is!" said the Fox. "How her feathers shine! What a beautiful form and what splendid wings! Such a wonderful Bird should have a very lovely voice, since everything else about her is so perfect. Could she sing just one song, I know I should hail her Queen of Birds."

Listening to these flattering words, the Crow forgot all her suspicion, and also her breakfast. She wanted very much to be called Queen of Birds. So she opened her beak wide to utter her loudest caw, and down fell the cheese straight into the Fox's open mouth.

"Thank you," said Master Fox sweetly, as he walked off. "Though it is cracked, you have a voice sure enough. But where are your wits?"

The flatterer lives at the expense of those who will listen to him.

The Fable of the Lion and the Hare
In ancient times, a ferocious lion lived in the forest, killing without remorse. The other animals were terrified. To stop the lion’s deadly hunts, some animals offered to provide him with food each day. Some animals would still die, of course, but the rest would live in peace. The lion agreed and enjoyed months of the easy life. One day it was the hare’s turn to present himself to the lion. Although small, the hare was very crafty.

“Lion, lion,” the hare cried out as he approached. “Help me, help me! Another lion is trying to eat me. But I am to be your dinner! You must stop him!”

Furious that another lion was trying to steal his food, the lion demanded, “Take me to the thief. I will make him pay for this mischief!”

The hare and the lion made their way through the forest, eventually reaching the deep well. There the lion looked down and saw his own reflection in the water. Thinking he had found the creature who tried to steal his food, the lion jumped down, ready to fight. Alas, the lion never came out of that well, and the animals lived in peace from that day on.

This text is provided courtesy of OLogy, the American Museum of Natural History's website for kids.

Caught in a Lie

Alexis tapped her pencil on her desk. Everyone in class was working on a math sheet, but she had already finished hers. Alexis was good at math. Her friend Brianna sat in the next row. Brianna was finished, too. She was leaning over and whispering to another girl, Kayla.

Alexis frowned. She and Brianna had been best friends ever since they were babies. They told each other everything. She and Brianna both agreed they didn't like Kayla. She was mean to some people and cared too much about how she dressed. So why was Brianna talking to her?

The bell rang. The teacher, Ms. Hunter, looked up from her desk. "Turn in your papers, class, and have a nice day!"

Alexis jumped out of her seat. She grabbed her backpack and ran to get in line. She tapped Brianna on the shoulder. "Hey, my brother's taking me to see a movie later," she said. "Do you want to come with us?"

Brianna looked down at her sneakers. "Um, I can't," she said. "My mom wants to take me clothes shopping tonight."

Alexis was disappointed. "That stinks! Maybe we can go next week, then."

"Sure, maybe," Brianna said.

Alexis's older brother, Ryan, picked her up from school in his car. He watched her after school sometimes while their parents worked. Ryan took Alexis for some take-out Mexican food. Then they went to the movie theater.

"I want to see that movie about the talking dog," Alexis said, as they walked into the lobby.

"Can't we see the one about the car race?" Ryan asked. Then he noticed something. "Hey, there's Brianna!"

Ryan pointed to the popcorn counter. He was right. Brianna was there—standing next to Kayla! Alexis was stunned. Why did Brianna lie to her?

Our New Old House

Boom!

A clap of thunder made the old house shake. Ramon huddled under the covers of his bed. A jagged streak of lightning lit up the room. I hate this house, Ramon thought. It was his first night in his new home. The house had belonged to his grandma. It was very, very old. Paint peeled off of the walls. The wood boards of the floor creaked when you walked on them.

The house looked really creepy at night, Ramon thought. The trees outside his windows looked like monsters in the dark. Their branches looked like long arms. They waved when the wind blew. The storm made everything extra creepy. Ramon hated storms anyway. But it somehow seemed worse in the old house.

Ramon pulled the covers off of his head. The thunder became quieter. Ramon drifted off to sleep. When he woke up, sun was streaming through his window. Ramon yawned and climbed out of bed.

The trees outside didn't look like monsters anymore. The branches had nice green leaves growing on them. Ramon could see a bird sitting on one of the branches. It chirped a happy song.

Ramon walked to the window and looked outside. It looked pretty nice in the daylight. A bubbling stream cut across the yard. A tire swing hung from one of the trees. A garden of flowers grew next to the tree. Butterflies flew around the flowers. "Maybe living here won't be so bad after all," Ramon said.

The High Dive

The summer sun warmed the cement under Alisa's bare feet as she walked around the edge of the town pool. Her friend Maria was waiting for her by the diving boards. "Come on, Alisa," Maria said. "Let's jump off the high dive."

Alisa looked at her with wide eyes. "The high dive? No way." "Don't be scared," Maria said. "It's really fun." Alisa walked away and sat on a bench. "You go. I'll watch you." Maria shook her head. "You don't know what you're missing."

Alisa frowned and bit her lip. She watched Maria climb up the tall ladder. It made Alisa dizzy just imagining being up there. Alisa had been afraid of the high dive since she could remember. The thought of being up so high terrified her. She had never even tried to jump off.

Maria jumped off the board and then climbed out of the pool. She approached Alisa. "Come on, Alisa, you've got to try it. It's awesome," she said. Alisa looked up at the board. Maybe it was time for a new experience. "Okay," she said.

Alisa climbed up the ladder for the high diving board. When she got to the top, she gripped the rails tightly. The water down below looked far, far away. She wanted to turn back, to walk back down the ladder, but that would be way too embarrassing. There was only one thing to do. Alisa walked to the edge of the board and took a deep breath. Then she closed her eyes and jumped. A rush of excitement coursed through her as she plummeted into the pool. She emerged from the water, sputtering and splashing. "That was awesome!" she cried.

Fourth Grade Passages

Vikings & European Explorers – Shipbuilding and Warfare

Vikings were not only fierce pirates; they were also the best shipbuilders of their time. Their masterful wooden ships were perfect for warfare. They were faster and stronger than those of other cultures. Water travel was the primary means of transportation for the Vikings. Most of Scandinavia is surrounded by water. Hundreds of fjords cut into the coastline. The fjords gave the Vikings easy access to the ocean and helped make them a seafaring people.

Viking ships are easy to identify because of their unique shape. The aerodynamic design starts and ends with a tall point at either end. But what made Viking ships sail better than the rest was something they developed called a *keel.* A keel is a long, narrow piece of wood that runs along the bottom of a boat. Like a fin, it cuts into the water and helps keep the ship steady so it can go faster. The keel also makes it easier to steer the ship.

There were two types of Viking ships. One was used for trade and the other for warfare. Trading ships were wide and short, usually about 50 feet long. Warships were thinner but longer, from 65 to 95 feet. Viking ships could travel at great speeds with the help of the wind and a huge woolen sail on a mast at the center of the boat. Most vessels also had oars. Rowers helped the ship through rivers and narrow straits. The front end of a Viking warship often had a carving of the head of a dragon or snake. Vikings used their swift ships to rule the seas and terrorize people. When they landed on a coast they would sneak quietly onto land so they could attack their victims by surprise.

Introduction to Owls

Owls are birds of prey. That means they hunt and eat other animals. Eagles, hawks, and falcons belong to that group too. But unlike most other birds of prey, most owls are nocturnal—they are active at night. Their nighttime activity, quiet flight, and strange calls have made them the subject of many stories.

There are more than 200 different kinds of owls all over the world, and North America is home to 19 of them. You may catch a glimpse of one along the side of a highway or hear it call from a tree in a city park or your backyard. The Great Horned Owl is found in every state in the United States except Hawaii. The giant of the owl family . . . in North America is the Great Gray Owl, which stands more than two feet high. The smallest is the Elf Owl—it's only about five inches tall. There is one thing all owls have in common: They are all excellent predators.

Learning about owls is an exciting adventure, and there are plenty of ways to do it. Some folks listen to recordings of owl sounds. Some watch live owl cams online while others see owls at nature centers. You can read books about owls and learn about them on the Internet.

SummerReads: Extreme Trees – Clone Trees

Most trees start from a seed. Some seeds like that of the double coconut tree are huge. The seed of the double coconut tree weighs up to 50 pounds. The seeds in an apple are much smaller than that of a coconut but even a single seed can produce an apple tree.

The quaking aspen is a tree that produces seeds but it can also reproduce through its roots. The roots of the quaking aspen spread out underground. In places where the soil and sunlight are right, a root sends out a stem. This stem forms a new quaking aspen. Over time, the roots of the new quaking aspen do the same thing.

When new trees grow seeds, they may differ from the parent trees in different ways. However, when a quaking aspen forms a new tree from its own roots, the new tree is exactly the same as the parent tree. These trees are clones of one another.

In Utah, there is a grove of quaking aspen trees that is called Pando. Pando means "I spread" in Latin. Today, there are 47,000 trees in the Pando grove in Utah. Each tree is a clone of a quaking aspen tree that started as a seed 80,000 years ago.

You probably won't see a tree this summer that is a clone of a tree that began 80,000 years ago. But look at the trees in your neighborhood. Many were growing long before you were born. What needs to happen so that they will be still be growing many years from now?

SummerReads: Mountains – Higher and Higher

About one fifth of the Earth's land area is made up of mountains. Each of the seven continents has mountains but their heights differ. The highest mountain is on the continent of Asia. Mount Everest in southern Asia is almost 30,000 feet high.

Many different kinds of plants and animals can be found on the same mountain but at different heights. The lower regions of mountains are often homes for many different kinds of plants and trees and of a variety of birds and animals. The higher you climb on a mountain, the cooler it gets and the growing season is shorter. Because food is harder to find, there are fewer kinds and smaller numbers of animals. Larger animals, such as elk and mountain goats, have to be able to climb steep mountain slopes.

The top of a very high mountain is usually in the alpine region. The alpine region is above the treeline, which means that trees can't grow in this area. The soil is bare and rocky. Snow stays on the ground for much of the year. Plants that grow in the alpine region, like grasses and mosses, are small but strong. The animals that live high on mountains tend to be small, too. Small rodents and rabbits can get by without as much food as bigger animals that live lower on the mountain.

A Small Life

It is not a bad life. I am warm and well fed. But my world is small. It's just 18 inches from side to side and back again. The floors are covered with bits of wood. I like the smell. The walls, well, they're not really walls. I can see through them. They're made of wires. I suppose most

everyone would call this a cage. But to me, it’s home. I have an amusement park ride in my little room. But there is no bed – just the floor. My kitchen is made of little plates that are attached to the wire walls.

You may have guessed by now that I am quite small. Actually, I am large for a hamster, but smaller than most members of this family, except the goldfish. That fellow is really little. His cage has glass walls. I don’t know what he does for fun.

The cat eyes me now and then, but has given up on getting a mouthful of me. When I’m tired of spinning on my running wheel, I like to drive him crazy. First I rattle something. The noise perks him up. Then I run around. Sometimes I stick my tongue out. That really sends him around the bend.

Yes, life is good. I wish, however, that I had more say about coming and going. Often, people just lift my whole house without asking if it’s all right with me. They are trying to be nice, I know. But when they pick up my abode and carry it around--well, it’s like an earthquake or something. Up and down! Side to side! Don’t they realize that I have feelings! Especially dizziness. I hang on for dear life and just hope that the journey is a short one.

A Very Special Place

When Lily needed a place to think, she headed to the old house. It was built back in the 1600s. A guard stood outside the iron fence that separated the wooden house from the apartment buildings around it. He always smiled at Lily when she visited the house. It was a special place to him, and he knew that it was special to her, too.

To the side of the house, there was a huge tree--an oak. People said that the man who built the house had planted it when he arrived in America from Holland. So, the tree was about 400 years old. Or not. (Some people said even an oak wouldn’t last that long.) Lily didn’t care. Its highest branches danced below a third-story window of the apartment next door. The tree cast a lot of shade. It always took her eyes a few minutes to adjust. Even on hot July city days, the space under the tree was cool.

Lily often brought a book with her. And a flashlight. There, she could read and imagine anything. She could pretend that the ants walking up the bark of the tree were knights marching off to battle. When a breeze blew the branches, she could peek up at the sky. Then she pretended that she was in outer space and that the blue was Earth. Once, a squirrel came right up to her and sat on her backpack. She found a potato chip bag in a pocket and opened it. Then she passed a chip to the squirrel. She thought it would run away. But the squirrel stayed there, holding the chip in its tiny hands, and ate it.

An Upset

The Hawks were in last place. Everyone agreed: they were the worst players in the whole league. Every other team had scored at least one win this season. But the Hawks were losers.

It was strange. Even though they never won, Taylor loved to play. He got along well with his teammates. Their coach was fun. He never yelled at them. A lot of the other coaches would scream and turn red. And they were the winning team! Why were they always so mad?

This was the final game of the season. As usual, the Hawks had gotten to the park early. They had something to eat. They kicked the ball around just for fun before the game. They had a large cheering section. Their parents lined the field. Some of them shouted, “Good job!” when a foot met a ball. When the goalie blocked a score, everyone cheered. Some parents ran onto the field. They looked silly, but they were having a good time.

The other teams did not have to work very hard to beat the Hawks. They took chances. They pretended to miss easy kicks. They sat down, waiting for the Hawks to move. They spun in circles to show they could do anything and still win. The Hawks tried to ignore their antics.

Then something happened. The other team missed an easy goal. The Hawks made two goals, one right after the other. A player on the other team slipped and fell. Taylor lobbed the ball halfway across the field. Another goal! The whistle blew. No one could believe what happened: The Hawks had won 3 to 1. As they left the park, they carried Taylor on their shoulders. It was a day none of them would ever forget.

The Park

Julie’s heart was pounding hard. She was in a tunnel. There were lights up ahead, but around her, everything was dark. She could hear music. It sounded like a carousel. She remembered being in this park before. It was about three years ago. She’d come here on a class trip. It was familiar. But it was strange, too.

Up ahead of her, she saw something moving. A horse? It was very odd. It was purple, with a golden mane. Something was definitely wrong. Nothing made any sense. She heard the sound of laughter behind her. Turning around, she was relieved. There was a group of children. They were wearing long flowing robes. They spoke in a language Julie had never heard before.

She went up to one of the girls in the group. She asked, “Where am I? Can you help me?” But the girl kept moving with her group. She didn’t even seem to see Julie. Then the girl ran ahead and mounted the purple horse. The other children trailed behind.

Julie kept walking. She was trying to remember the path out of the park. It was so long ago. She didn’t know if she could find her way back.

She was scared. Then she heard a voice. It was her mother’s. There was a bright light. “Wake up, Julie!” her mother said. She pulled back the curtain. The sun poured into Julie’s room. It was morning.

A New Home

Patience looked around; on every side of the trail were trees. They were so tall they blocked out the light. When she looked up she could see only a few bits of blue. Ahead, branches almost blocked the trail. The trail was nothing more than a narrow path. The ground was hard and rough. She stubbed her toe on a rock poking out from the dirt. The ground here seemed full of stones and rocks.

Her long skirt did not make it easy to move. She walked behind her sister. Her father led the way. Her mother carried the baby. Her brother pushed a cart carrying their belongings. They had to stop often to unload the cart. Then they would carry the things they unloaded up a hill. Or they would lift them over a fallen tree. Then her father and brother would lift the cart over the obstacle and push it ahead. Then one by one they would reload the cart. The things they carried were few. There were some pots, quilts, a few spoons, cloth, a bag of seeds and some tools.

They had left the coast several weeks before. There the land had been cleared. Open fields stretched down to the sea. Flowers in gardens tipped their faces to the sky. People lived in snug houses. But that comfort was left behind when they entered the dark woods.

Patience couldn't wait to get to the end of the trail. It would be home. She knew several other families had gone on ahead. They were clearing the land and bringing light to the center of the forest. Patience knew it would be a very small patch of open land. It could take forever to clear this stony land. She hoped there would be time.

SummerReads: Bikes & Boards – Bicycles

The first bicycles did not look like the bicycles of today. Their frames and wheel rims were made of wood or solid metal that made them heavy and hard to ride. One of the early bicycles had a very big wheel in front and a very small wheel in back. People in England named it the penny-farthing because it reminded them of two English coins. The penny was a big coin and the farthing a tiny coin.

The pedals on a penny-farthing were joined to the hub of the big front wheel. That meant that the rider had to sit almost on top of the front wheel to reach the pedals. A bump on the road could send a rider flying over the front of the bicycle. Many riders got hurt and even died. These bicycles were just for adults, usually men.

A little over 100 years ago, three inventions led to bicycles like those of today. First, the use of chains and gears meant that pedals could be joined to the frame rather than to the wheel. Wheels could now be the same size and riders could be seated lower and further back on the bicycle.

The second invention was a way of treating rubber. This made it possible to have air-filled rubber tires. Air-filled rubber tires made a smooth ride, unlike that on wooden or metal tires.

Third, the invention of hollow, metal frames meant that bicycles were no longer as heavy. These changes made bicycles safer and easier to ride. Now bicycles could be for everyone—men, women and children.

Buried Treasure

Dig this! Archaeologists in Egypt are digging up and restoring an ancient boat. An archaeologist studies old objects to learn about the past.

The 4,500-year-old vessel, or boat, is buried in an underground room next to the Great Pyramid. Egyptians built the Pyramids thousands of years ago as burial spots for their pharaohs, or kings.

This boat is the second found near the Great Pyramid. A similar boat was dug up in many pieces in 1954. When the vessel was rebuilt, it stretched about 142 feet long. The boat is on display in a museum above the spot where it was discovered.

Some experts think the boats were used to transport the body of the pharaoh Khufu, who built the Great Pyramid, after his death. Others don't think that theory holds water. They are not sure the boats ever got wet. New research on the second boat could help scientists figure out the boats' purpose.

"In Egypt, almost everything real had its ... meaning ... in the spiritual world," says scientist John Darnell. "But there's a lot of debate as to whether these vessels ever were used or not."

Experts will begin removing the boat in about 600 pieces and try to rebuild it. Until then, tourists can view images of the buried boat taken by a camera inserted through a hole in the underground room's ceiling.

Earth 2072

The year was 2072. Beth touched the start button. The voice came on. "Good morning, Beth. Log into lesson number 9."

Beth liked school. She liked sitting in her study pod. Today's lesson was on history. She waited for the other children from around her home planet to sign on. There was also a visitor. It was a girl from station 54. It was near Mars. That was a place she really wanted to visit. Her parents promised that they could go there soon.

She signed on. She loved to hear the chatter on the line.

"Did you catch the meteor shower last night?" Nate asked.

"How could I miss it!" Jay interrupted.

"Amazing! We had to move our ship to get out of the way!" Beth said.

The robot started the lesson.

"Up until just 40 years ago," it said, "children learned together in school buildings. They worked together with a human teacher. But today, we are far ahead of the old ways."

The robo-teacher kept talking. Beth began to think what it would have been like in the old days. She had never met another child face to face. Would it be strange to be in a room with another person?

Comprehension Questions

Second Grade Passages

Lily's Timeline

1. The first event is written on the left. (T)
2. Lily's first event was learning to walk. (F)
3. Lily didn't want to add any pictures to her timeline. (F)

At the Lake

1. Summer weather warmed the lake. (T)
2. Fog is small drops of water in the air. (T)
3. Layla thinks the lake is only fun in the summer. (F)

Summer Reads: Moths – Silkworms

1. The cocoon is made of many small threads. (F)
2. Silkworms are mostly found in Africa. (F)
3. The thread of the cocoon is used to make clothing. (T)

The Volcano That Keeps Erupting

1. Hot, melted rock is called lava. (T)
2. All volcanoes are tall like mountains (F)
3. Kilauea has erupted many times. (T)

The Time Machine

1. To activate the time machine, all you had to do is say the date out loud. (F)
2. Eric landed in a desert. (F)
3. Eric saw a T-Rex. (F)

A Clean Park

1. Tyler had never noticed the garbage in the park before. (T)
2. Tyler and his friends cleaned the park all day. (F)
3. Tyler felt better after he helped clean the park. (T)

Martin's Birdhouse

1. Drawing pictures can help you share ideas. (T)
2. Martin and Diego built the birdhouse together. (T)
3. This was Martin's first time building a birdhouse. (F)

I Want to Buy a Computer Game

1. Luis's mom gave him $35. (F)
2. Luis saved money by buying the game on sale. (T)

3. Luis’s mom was proud of him for saving his own money. (T)

Oh Nuts!

1. Daisy helped Chestnut collect nuts in the fall. (F)
2. Chestnut shared the nuts with Daisy. (T)
3. Daisy promised to help Chestnut collect nuts next fall. (T)

The Rocky Mountains

1. The Rocky Mountains are the longest mountain range in North America. (T)
2. The highest peak in the Rockies is Mount Rainier. (F)
3. Many types of animals live in the Rockies. (T)

A Time of Dust Storms

1. A drought is a long period without snow. (F)
2. Children wore masks so they wouldn’t breathe in the dust. (T)
3. Plants help keep the soil in place. (T)

When TV Became Colorful

1. Television has always been in color. (F)
2. Televisions used to have a dial to adjust the amount of color. (T)
3. Most people in the U.S. have color televisions. (T)

Third Grade Passages

Return of the Bear

1. A habitat is a place in nature where an animal lives. (T)
2. The Grizzly Bear population has declined since the 1980s. (F)
3. No Grizzly bears are protected by the Endangered Species Act. (F)

What is Light?

1. Gases from the Sun give off heat and light. (T)
2. Light can travel around the Earth one time every second. (F)
3. Long light waves carry the most energy. (F)

Summerreads: Lakes – The Great Salt Lake

1. Most lakes are saltwater. (F)
2. The Great Salt Lake has many streams and rivers that flow out of it. (F)
3. Only pure water can evaporate. (T)

Trouble in the Amazon

1. Trees help clean the air. (T)
2. The Amazon is the second-largest rainforest in the world. (F)
3. Humans contribute to the Amazon shrinking. (T)

Australia's Wonder: The Great Barrier Reef

1. The Great Barrier Reef can be seen from outer space. (T)
2. The coral is mostly white. (F)
3. Pollution and litter hurt the Great Barrier Reef. (T)

The Northern Lights

1. Particles from the Moon cause the Northern Lights. (F)
2. Different gases give off different colored lights. (T)
3. This light show only happens at the North Pole. (F)

Sunflowers

1. Sunflowers are made up of two kinds of flowers. (T)
2. Only humans eat sunflower seeds. (F)
3. The stems of sunflowers are smooth. (F)

The Fox and the Crow

1. The crow was holding a piece of bread in its beak. (F)
2. The fox tricked the crow. (T)
3. The crow wanted to be called the Queen of Singing. (F)

The Lion and the Hare

1. The animals were friends with the lion. (F)
2. The hare played a trick on the lion. (T)
3. Another lion was actually stealing the lion's food. (F)

Caught in a Lie

1. Alexis was happy that Brianna had made a new friend. (F)
2. Brianna lied to Kayla. (T)
3. Kayla is a kind person. (F)

Our New Old House

1. Things in Ramon's house looked spooky at nighttime. (T)
2. Thunder can be very loud. (T)
3. There is a pond in Ramon's backyard. (F)

The High Dive

1. Alisa had always wanted to jump off of the high dive. (F)
2. Maria forced Alisa to jump off the road. (F)
3. Alisa was glad that she overcame her fear. (T)

Fourth Grade Passages

Vikings and Europeans Explorers

1. A keel is a long, narrow piece of wood that runs around the side of a ship. (F)
2. Trading ships were usually longer than warfare boats. (F)

3. Viking ships could travel at great speeds with the help of a large motor. (F)

Introduction to Owls

1. Being nocturnal means an animal is mostly active during the daytime. (F)
2. The Great Horned Owl is found in all 50 states. (F)
3. The Elf Owl is the smallest owl in North America. (T)

Summer Reads: Extreme Trees

1. The quaking aspen can only reproduce through seeds. (F)
2. Quaking aspens create clones of the themselves. (T)
3. Pando means “identical” in Latin (F)

Summer Reads: Mountains

1. Mount Everest is the tallest mountain in the world. (T)
2. The higher regions of mountains have more plants and animals than the lower regions. (F)
3. The alpine region of a mountain is below the tree line. (F)

A Small Life

1. The hamster likes to tease the cat. (T)
2. The hamster wishes he could communicate with his family. (T)
3. The hamster likes his cage. (T)

A Very Special Place

1. Lily likes to sit under the tree and paint. (F)
2. Lily gave the squirrel an acorn to eat. (F)
3. The guard didn’t like that Lily visited the house. (F)

An Upset

1. The Hawks were a baseball team. (F)
2. Even though the Hawks never win, the team still had a lot of fun playing. (T)
3. The Hawks won their final game of the season. (T)

The Park

1. Julie was having a strange dream. (T)
2. The girl in the group of children helped Julie find her way home. (F)
3. Julie was lost. (T)

A New Home

1. Patience and her family were walking through a forest. (T)
2. Patience’s family was travelling with a large load of their belongings. (F)
3. The family has been travelling for year. (F)

Summer Reads: Bikes & Boards

1. Some of the first bicycles were made out of wood. (T)
2. Bicycles were designed mostly for children. (F)
3. Today, bicycles are heavier than they used to be. (F)

Buried Treasure

1. The Egyptian Pyramids were burial spots for animals. (F)
2. Scientists debate over the original use of the boats. (T)
3. Archaeologists are trying to dig up the boat all in one piece. (F)

Earth 2072

1. This is likely a true story. (F)
2. Beth has been to Mars. (F)
3. Beth watched the meteor shower last night. (T)
