# Supplementary material for: Annotating digital text with phonemic cues to support decoding in struggling readers
Source: PLoS One. 2020 Dec 7;15(12):e0243435. doi: 10.1371/journal.pone.0243435 (PMC7721157; doi:10.1371/journal.pone.0243435)
Supplement: Attachment — Submitted filename: Response To Reviewers.pdf [file pone.0243435.s006.pdf]

We would like to thank the reviewers once again for their thoughtful and insightful comments on this study. We have made further revisions and agree with the reviewers' points that these changes will make this manuscript suitable for publishing. The reviewers **comments are below in black** and our **responses and specific actions are noted in blue**.

Reviewer #1: I would like to thank the authors for the changes they have made in this version.

The authors made an effort to address my comments in the previous round of review. For example, I commented that the study should refer to the extensive scientific literature on what works for struggling readers in terms of best practices as well as existing studies on embedded digital support tools. While this version provides some literature review of existing studies on embedded digital supports, it still doesn't provide any literature review on research-based best practices that the authors refer to such as (e.g., phonemic awareness, phonics etc.).

We thank the reviewer for this added clarification on the missing references in our literature review section. We have added necessary citations to the statement above " (e.g., phonemic awareness, phonics etc.)" on page 4. Beyond explanation of our phonemic cue in the context of other embedded supports, we believe a more thorough review of specific instructional techniques for phonics/phonemic awareness is beyond the scope of this manuscript. In this revision we do, however, refer the reader to the most comprehensive review papers and meta-analyses. Additionally, explanation of the focus of the app is provided in the App design section (page 9-10) detailing its use in the context of broader phonics curricula.

The section of the introduction now reads:

"Decades of scientific research into the behavioral and neural mechanisms of literacy learning has led to the development and testing of effective intervention programs for struggling readers, and established comprehensive guidelines and best-practices for implementation of an effective curriculum [2,13–15]. Unfortunately, these evidence-based practices (e.g., phonemic awareness, phonics) are largely not being incorporated into the current technological boom. For example, a consistent finding in the intervention literature is that children with dyslexia benefit from direct instruction in phonological awareness and curricula that make clear links between orthography and phonology whereas children with stronger reading skills can often infer grapheme-phoneme correspondences without direct instruction (for review of the extensive literature on the importance of phonics/phonemic awareness see [16–23]). " (P4, L8-17)

Additionally, in this version, they referred to 3 meta-analysis (9, 16, 17) that demonstrate promise for digital solutions in the context of literacy. However, all these 3 studies were conducted pretty much by the same authors. It looks as if the authors published the same or similar meta-analyses in three different publications. If that is the case, I would advise referring to only one of the meta-analysis rather than all three and cite some other existing studies on embedded digital support tools. Rather than explaining each study, synthesize results into a summary of what is and is not known, identifying areas of controversy in the literature.

Although the meta analyses cited have very similar authorship, they provide slightly different perspectives on the literature that we believe provide a better picture of the state of research in this area. However, we do agree that the first (Cheung & Slavin, 2011) substantially overlaps with the Slavin, et al., 2011 and have removed the first from the citation. The primary difference in contribution between the Slavin, et al., 2011 and the Cheung & Slavin, 2013 is that the latter provides a specific context for struggling readers whereas the former applies to all elementary school youth. We believe that both perspectives are highly relevant and have amended the section to the following with some added synthesis:

"Recent metanalyses demonstrate much promise for digital solutions in the context of literacy, yet also describe the multitude of ways that technology is an inappropriate substitute for many aspects of pedagogy [24,25]. Namely, these meta analyses demonstrate that technologies focused on supplementing what is provided in the evidence-based classroom (i.e. explicit phonics), rather than restructuring at the classroom level, have demonstrated the most promise in the digital landscape. These findings, however, should be interpreted with caution as the authors further contend that the preponderance of studies in this area are characterized by small samples and poor study design [25]. " (P5, L3-10)

Reviewer #2: Review for PONE-D-19-34461R1: "Annotating digital text with phonemic cues to support decoding in struggling readers"

I would like to thank the authors for transparently addressing the raised points and providing a much-improved manuscript. Below is a list of mostly minor points which still require further attention or clarification. My only major concern relates to the data analysis and provided code.

Thank you

Introduction:

Regarding research questions (P6:L14-17) and hypotheses (P6:L17-23) none of the research questions contains exposure and you added the hypothesis of an intervention x exposure interaction to the second research question, although it would also be valid for the first one, and ultimately perhaps be a better fit with the third one. Is there a specific reason for that?

We thank the reviewer for identifying this point. We indicated an exposure effect in the second question only because we believed that children would require significantly more practice than the 2-week period to motivate effective use during connected text reading (question 1). As the second question related to single word decoding in a more "assessment" context we believed we would see more active use and, in extension, more effect of exposure.

Method:

Following up on your clarification of my mayor point 2 in the previous version of the manuscript, you added that at post-intervention only intervention participants saw the cues and control participants had cues disabled. Could you further clarify how the assessment looked at pre-test? Did both groups read without cues? Or was it already enabled for the intervention group? And did the assessment take place before or after the introduction training with the app, or was the pre-assessment done offline in a pencil and paper style?

We apologize for the confusion that was met in parsing our study design. The first sentence of the study design section indicates that *"all participants completed an initial, baseline session that collected all outcome measures using a normal text condition presented on a Kindle fire tablet without the Sound It Out cue" (L6-8)*. The training period for both groups (intervention and control) occurred after this baseline testing with the normal text condition as detailed in the remaining paragraph. All testing was completed on a Kindle Fire tablet. We have further adjusted the text to clarify this point.

Considering that grapheme-phoneme associations also depends on availability of high-quality phoneme representations, could you clarify whether the recordings you added to the app were judged for prototypicality, and whether children were instructed to play with headphones or not?

The recordings were created by a member of the study team (KL) and were judged to be typical examples of the vowel sounds by PD & JD. All three are native English speakers. Furthermore, the vowel sounds were played during the training period and kids were given the opportunity to indicate if a given recording was unclear. The following has been added to the manuscript for clarity on this point:

"The vowel sounds were recorded by a native English speaker with training in phonetics. The recordings were judged by the three native English-speaking authors to be typical examples of the given vowel sounds and, during the training period, participants were exposed to all the vowel sounds and were able to correctly identify the vowels." (P10, L20-P11,L2)

P7:L16: I assume classification as struggling reader happened within one year "prior" to participation?

Yes - the statement has been amended to the following: ***"The participants (19 females; 21 males) were classified as struggling readers based on a battery of behavioral measurements administered within one year prior to participation in the present study." (P8, L4-6)***

P8:L11: If ADHD was not an exclusion criterion could you provide numbers on how many children had this diagnosis?

Yes - the following statement has been added: ***"In our sample 12 children had a diagnosis of ADHD (6 Control, 6 Intervention)." (P9, L1-2)***

P11:L22: The instruction "When you come a word you don't know..." does not appear to be grammatical?

Thank you for catching this typo - we have amended the statement to the following: ***"When you come to a word you don't know, just look at the symbols and that should help you figure out the sounds that the blue letters make." (P12, L19-21)***

P13:L1-3: Given that you do not have logs for exposure and credit exposure based on the comprehension questions I wanted to point out that the comprehension questions are all True/False responses and therefore the probability of getting 2 or more correct replies by just guessing (and therefore being credited with the exposure) is rather high at 0.5. Did you consider to analyze these comprehension data for intervention effects as well?

We did look to see if there were any differences in practice between control and intervention participants, and found that there was no difference between the distributions of practice using an independent t test ($t = -0.592$, $p=0.558$).

P14:L7: Please avoid exponential notation for p-values and shorten to $p < 0.001$ according to common convention.

We have amended where applicable.

P14:L9: Could you clarify whether the Woodcock Johnson test measures timed or untimed decoding?

We have added clarification - the statement now reads: "Accuracy of real and pseudo word decoding was our primary outcome measure (number of words read correctly on each list akin to the Woodcock Johnson Word ID and Word Attack (both untimed measures))." (P15, L4-6)

Data analysis:

P8:L13: In the caption of Table 1 you say that you conducted independent t-tests. However, not all variables are normally distributed, and you must use the wilcox/mann-whitney test in these cases. It does not influence the fact that there are not differences between the groups though.

Thank you for pointing this out - I have performed the Wilcoxon signed-rank test on Age/Gender to conclude that all characteristics demonstrate no significant differences. The caption now reads:

"Demographic information for participants in the Intervention and Control groups. See Methods for descriptions of the individual characteristics. For each characteristic, the mean is provided with the standard deviation within parentheses. Independent t-tests - and Wilcoxon signed-rank tests for Age/Gender - performed demonstrate no significant differences across all characteristics."

Thank you for carrying out additional analyses with age and initial phonological awareness as covariates. Could you still add these models to the supplementary information and add a sentence in the results section indicating that this was done and yielded comparable results?

We have added the following to the manuscript to indicate that this analysis was performed with a parenthetical reference to a more in-depth explanation in the supplement:

"Due to the heterogeneity of our sample, we tested a model with added covariates for age and initial phonological awareness ability: model fit comparison revealed no benefit to the more

complex model and no significant main effects for the added covariates (S1 File)." (P18, L23 - P19, L3)

"Similarly concerned with the heterogeneity of our sample, we tested a model with added covariates for age and initial phonological awareness ability: model fit comparison and analysis of added fixed effects demonstrated no significant effect (S1 File)." (P20, L10-13)

The following has been added to the Supplement:

"Role of Age and Phonological Awareness as Covariates to Mixed Effects Models

Due to the heterogeneity of our struggling reader population in both reading ability and age, we performed an exploratory analysis of these results testing a model that adds covariates for participant age and initial phonological awareness ability (as measured using the CTOPP phonological awareness composite measure).

Model fits were compared using AIC/BIC values and revealed that in no case was the model with added covariates a superior model to the simpler model used in the manuscript. Looking at the results of these models, moreover, revealed no significant effects of age or phonological awareness. Interaction effects were unchanged from the simpler models reported in the manuscript.

The model fit workflow and code associated with this analysis can be found in the associated project repository on GitHub."

P15:L6-L15: Thank you for providing the code book which allowed me to have a look at the data myself. There are still mayor issues with the data analysis though. In the manuscript you state that you added independent random intercepts of time and participant - which would suggest (1|time) and (1|participant), in the Matlab code you have session nested within participants (1-session|participant) as well as a reading list random intercept (1|acc_Indicator). The former is not well specified though, because "1-" ignores the session part. It should be specified as (1+session|participant) or because the 1 is implied (session|participant). Was there a specific intention behind using "1-"? In any case, this changes the coefficients only minimally but changes the confidence intervals quite a bit. I'm also concerned that this is a very complex random effects structure for the little data you have and you might be overfitting. I would suggest to do model comparison (with AIC/BIC) to see which random effects structure is

required. Did you also check that model assumptions were fulfilled after fitting (normality and homoscedasticity of residuals?)

We thank the reviewer for drawing attention to this issue in our statistical model. We had in fact run model comparisons to determine the ideal random effects structure. The notation issue raised by the reviewer was due to incorrect documentation in a previous version of MATLAB. The best fitting model was, in fact, the simple one suggested by the reviewer with a random intercept for participant. We now provide a more detailed model fit workflow. For the word list data, we also added a random intercept for list number (1|acc_Indicator) because we found it to vastly improve model fit due to slight variations between word lists (though including or excluding this random effect does not impact the effects of interest). We have repeated all analyses using this random effects structure with no major changes to our main findings. We also add to our uploaded code checks for normality in residual distribution/heteroscedasticity. Analyses reveal that in all model fits there is a normal distribution of residuals and no evidence of heteroscedasticity.

The following has been amended to the manuscript:

"For each outcome measure, we fit an LME model with fixed effects of: (1) time (pre-intervention / post-intervention as a categorical variable); (2) group (intervention / control groups as a categorical variable); (3) the group by time interaction. The models included a random effect for participant, to account for individual variation in baseline performance. To account for differences between the individual, lab-created word lists, we added a random effect for word list to those models. " (P16, L6-11)

We have also amended the "Transparent Changes" document in our preregistration.

Furthermore, the statistics which are presented in the results section do only partially stem from the provided Matlab code. It is, for example, not apparent where the main effects of group and session (P16:L21-L22) come from and why they only have 78 degrees of freedom when these effects have 156 DF in the model. Please make sure to provide all code for results which you provide in the manuscript (also for possible post-hoc or correlation analyses as well as calculations of effect sizes).

The mentioned analyses were performed post-hoc, and for clarity have been changed to paired t-tests on subsets of the dataset to help illustrate the differences between groups

and provide perspective for the interaction results. Discussion of these results in the manuscript have been amended to clarify that distinction:

"For real-word decoding accuracy the group by time interaction was not significant (β=1.3, t(156) =1.923, p=0.056) indicating that the growth in the intervention group was not statistically different from the control group. Post-hoc, paired t-test analyses dividing the data at the group level revealed a significant increase in in the intervention group (t(19) = 3.75, p = 0.001) and a non-significant increase in the control group (t(19) = 1.10, p = 0.285)."

"For pseudo-word decoding accuracy the group by time interaction was significant (β=3.175, t(156)=2.99, p=0.003) with the intervention group showing significantly greater improvement than the control group (a threshold of 0.0125 was defined in the preregistered report to adjust for multiple comparisons). At pretest, despite randomization, the intervention group by chance had lower scores than the control group: this is evidenced by the significant main effect of group in the mixed effects model. Post-hoc, paired t-test analyses at the group level revealed there was a significant increase in the intervention group (t(19) = 2.176, p = 0.042) and a small but non-significant decrease in the control group (t(19) = -1.149, p = 0.265)."

"For word reading accuracy the group by time interaction was not significant (β=0.014, t(65)=1.1, p=0.275). Post-hoc, paired t-test analyses dividing the data at the group level revealed a non-significant increase in the intervention group (t(15) = 2.325, p = 0.035) and a non-significant increase in the control group (t(17)=0.518, p = 0.611). [JY1] ***For word reading rate there was a non-significant group by time interaction (β=0.014, t(64)=0.368, p=0.714). Post-hoc, paired t-test analyses at the group level revealed a non-significant increase in both the intervention (t(14)=1.468, p = 0.164) and control groups (t(17)=1.000, p = 0.331)."***

We have also ensured that these and other analyses are available in the public repository.

With the pseudoword model (P17:L3-L8) there is also the issue that it describes a big and significant difference between the two groups at pretest (with the intervention group scoring 6 words lower) while at post-test the two groups are at the same level again. This is not mentioned in the results section and sheds a different light on the sole significant effect.

This is made worse by plotting only pre-post differences and the use of bar charts to represent the data as it distorts the perception of observed values and draws attention to unimportant aspects (i.e. bar height rather than difference between means). See, e.g.: https://doi.org/10.1371/journal.pbio.1002128

Consider using dotplots or boxplots of the raw pre-post data with an indicator of the means.

This is a very important point and we have added the following to the results section to add clarity and transparency:

"For pseudo-word decoding accuracy the group by time interaction was significant (β=3.175, t(156)=2.99, p=0.003) with the intervention group showing significantly greater improvement than the control group (a threshold of 0.0125 was defined in the preregistered report to adjust for multiple comparisons). At pretest, despite randomization, the intervention group by chance had lower scores than the control group: this is evidenced by the significant main effect of group in the mixed effects model. Post-hoc, paired t-test analyses at the group level revealed there was a significant increase in the intervention group (t(19) = 2.176, p = 0.042) and a small but non-significant decrease in the control group (t(19) = -1.149, p = 0.265)." (P18, L3-10)

We have also added violin plots to the Supplemental information (S1 Fig) to provide more detailed visualizations of the data.

P16:L17: I understand that due to unreliable usage statistics the correlation analysis was the best you can do, but this is not a valid approach for a prediction analysis. It's best to be transparent about the unreliable data by adding a few sentences from the response letter to the statistics part of the methods section and also bring this up in the limitations. Or remove this analysis altogether after mentioning that this part of the data collection did not work as intended.

Thank you for this insight. We agree that it is important to be more transparent about this limitation and have amended the statistics section to read:

"Due to issues collecting reliable usage statistics for the at home reading practice, prediction analyses were not appropriate. Instead, post-hoc correlation analyses were performed using the Pearson correlation coefficient between post-pre difference scores and the three subject characteristics collected at baseline: age, WASI-II and the CTOPP-2. This analysis differs from that described in the preregistration due to the small number of reading variables collected and inability to collect robust measures of exposure, making methods of dimensionality reduction not appropriate." (P16, L14-20)

We have also amended the Discussion section to specify Correlation rather than Prediction analysis and draw the reader's attention again to the limited nature of this dataset:

"Correlation analyses, moreover, were inconclusive but suggested that the tool may benefit those participants who are younger and/or have lower phonological processing scores (see

Results). However, these results were not significant (after multiple comparison correction) and, due to unreliable practice data (see Statistics), insufficient to support any conclusions regarding the relationship between subject characteristics and benefits conferred by phonemic cues" (P21, L11-16)

We have also added the following to the limitations section:

"First, future studies should more efficiently, and quantitatively and qualitatively, monitor practice adherence and cadence at home to better explore the relationship between exposure and reading-related measures." (P24, L7-9)

Results:

P16:L21: You are using ß (latin sharp s) instead of β (greek beta), as well as a p-value with 5 decimals.

We have amended accordingly.

P18:L1-3: Repeated use of "pronounced". Furthermore, the effect was not "particularly pronounced" for the pseudoword decoding, but it was exclusively there.

We have amended the paragraph to read:

"These findings show that without the constraints of time during testing, there was a beneficial effect of access to the phonemic cue for single word decoding. This benefit was observed in the case of pseudoword decoding where children were asked to pronounce novel words in isolation. Moreover, prediction analyses suggest that this effect is more pronounced for those participants with more significant impairments in phonological processing and lower IQ (though these effects did not surpass our adjusted significance threshold of $p < 0.0125$). "

P18:L3-5: Please present the statistics if you refer to effects which suggest something. Also in the Matlab script.

Now when we say suggest, we provide relevant statistics that suggest this idea. In cases that we do not have a statistic, but suggestion of a hypothesis in future work, we note that in the paper.

P19:L3: Significance and effect sizes are completely unrelated. We can observe giant yet non-significant effects, as well as extremely small (and thus irrelevant) yet highly significant effects.

We have amended the statement to read: ***"Further, data reflected effect sizes of d = 0.36 for accuracy and 0.26 for rate."***

Discussion:

P19:L22-P20:L4: I find this part a bit misleading as it jumps back and forth: things are inconclusive; a relation between pretest phonological awareness and intervention outcome is mentioned for which no statistic is provided in the results; two mentions that after p-value adjustment there were no effects left, but it is still discussed that these effects might suggest something. Especially at the start of the discussion there should be a clear summary of what was found first and then one can discuss what the presence and absence of effects might indicate.

We thank the reviewer for this comment and agree that the start of the discussion section is not as clear as it should be. We have amended the first paragraph to provide a better summary of the findings before proceeding on to our interpretation. Furthermore, we have amended the wording to make clear that the relationship between phonological awareness and age is related to the correlation post-hoc analysis in the results section.

"Using a RCT design, we tested the hypotheses that struggling readers could leverage a phonemic image cue placed below the vowels in digitally presented text to improve reading accuracy for isolated words and connected text, and that this benefit would be more pronounced for those readers with lower performance on measures on phonological processing. Data collected after a two-week period of unsupervised (but digitally monitored) practice demonstrated that struggling readers could read more complex words using the tool: compared to the control group, the intervention group showed a significantly larger improvement in decoding accuracy specifically for pseudo-words. As depicted in the results, this benefit did not extend to real words or either measure related to connected-text reading (accuracy and rate).

Although there was no benefit, stable performance on measures of connected text read was observed for all participants with no significant difference between groups. The lack of benefits for connected text reading might reflect the limited training period or the increased cognitive demands of a novel approach to reading. These are important questions for future studies as generalization to connected text is of key importance.

Correlation analyses, moreover, were inconclusive but suggest that the tool may benefit those participants who are younger and/or have lower phonological processing scores (see Results)"

P20:L16: [...] highly inconsistent grapheme-phoneme mapping "of English" [...]

The sentence now reads: "We focused on vowels because, in English, the highly inconsistent grapheme-phoneme mapping is a major hurdle for struggling readers."

P22:L3-L5: Throughout the manuscript there are sentences which use many commas and therefore appear encapsulated and make it unnecessarily hard for the reader to grasp the most relevant information. In this case you can straight out write that there is an improved decoding of pseudowords and remove the subordinate clause. As this is the only comment regarding writing style, I also wanted to note that there appear to be a lot of double spaces in manuscript. Content wise it could be highlighted here that this improvement appeared after being trained with the app for (only) 2 weeks.

Thank you for this feedback - we certainly want to make sure that the manuscript is clear and readable. We have deleted the subordinate clause in that sentence and have removed double spaces.

P23:L5-L13: This is an interesting observation. They appeared to have learned to make use of the cues, but this disappears in the testing situation. I feel it's quite relevant to understand this better. Given that there is no performance penalty it seems like they might be able to ignore the cues altogether? One could take a more qualitative approach and investigate how children use it in these different scenarios or alternatively add an assessment mode into the app itself which blends with the intervention?

This is a great point and represents a significant hurdle for any pedagogy to motivate a challenged reader even when they know it works/helps. We had a constant debate about how to measure use while reading and will contend that it is possible the children were ignoring the symbols altogether. During testing the children were reminded quite frequently to utilize the symbols, but as we discuss in the manuscript: 'Either due to the limited practice period, limited supervised practice, or conflict with existing strategies children use when approaching challenging words,' they didn't feel as motivated to adopt the cues. We agree that future studies should work to collect better measures to piece this apart and have added the following to that paragraph in the discussion:

"Future studies should incorporate qualitative and metacognitive methods to identify factors and circumstances that encourage struggling readers to adopt a novel strategy." (P23, L22 - P24, L2).

P23:L23: Given that parents also got a brief introduction to the app and you come back to it in the discussion it might be worth to recommend measuring parental involvement in future studies?

We agree. The paragraph now reads:

"Albeit promising, these results should be interpreted cautiously: Our power analysis indicated that we only had sufficient power to detect relatively large effects and many of the analyses were likely underpowered. Thus, future studies are needed with larger sample sizes to provide more conclusive results. Moreover, two additional points merit further investigation. First, future studies should more efficiently monitor practice adherence and cadence at home to better explore the relationship between exposure and reading-related measures. Second, given the short intervention period, we did not examine generalization to reading improvements without the cue and across different aspects of skilled reading. We only investigated whether the cue could be effectively used to decode more complex words. Thus, examining long-term learning effects and generalization to a variety of different contexts, as well as the role of parental involvement/participation is an important future direction."

Summary:

In summary, the manuscript has much improved and will be a relevant addition to the field, but the provided analysis and code are not yet up to the required standards.

We thank the reviewer for these relevant and meaningful recommendations for our manuscript. We believe the work is now much stronger for it.
