# Supplementary material for: Annotating digital text with phonemic cues to support decoding in struggling readers
Source: PLoS One. 2020 Dec 7;15(12):e0243435. doi: 10.1371/journal.pone.0243435 (PMC7721157; doi:10.1371/journal.pone.0243435)
Supplement: Attachment — Submitted filename: Response to Reviewers.pdf [file pone.0243435.s007.pdf]

We would like to thank the reviewer and have provided responses for each concern. The reviewers **comments are below in black** and our **responses and specific actions are noted in blue**.

Reviewer #2: Thank you for your detailed answers and providing a, once more, much improved manuscript. I would suggest incorporating the following minor changes prior to publication:

You now mention the differences in pseudoword decoding at pre-test in the discussion, but these are not yet reported in the results section. Please add the relevant statistics to the results section, if possible with effect size.

The results has been added and now reads:

"At pretest, despite randomization, the intervention group by chance had lower scores than the control group: this is evidenced by the significant main effect of group in the mixed effects model (β=-6.2, $t(156) = -2.65$, p=0.009)."

I would suggest to add "small scale RCT" (on P21:L11) and/or "small scale proof of concept" (on P26:L17).

We have amended the text to reflect your suggestions. Those lines are now amended to read:

"Using a small scale RCT design, we tested the hypotheses that …", and "In aggregate, these findings represent a small scale *proof-of-concept* for this …"

P24:L2-5: The repeated use of "future studies" reads a bit awkward. I'd suggest to slightly rephrase.

We agree and have amended to the following:

"Also, as there was a significant difference at pre-test for our sole finding with pseudo word decoding, future studies are needed to rule out the role of regression toward the mean and possible ceiling effects. Thus, future work is needed with larger sample sizes to provide more conclusive results. Moreover, two additional points merit further investigation. First, future experiments should more efficiently, and quantitatively and qualitatively, monitor practice adherence and cadence at home to better explore the relationship between exposure and reading-related measures."
