# Supplementary material for: Annotating digital text with phonemic cues to support decoding in struggling readers
Source: PLoS One. 2020 Dec 7;15(12):e0243435. doi: 10.1371/journal.pone.0243435 (PMC7721157; doi:10.1371/journal.pone.0243435)
Supplement: Attachment — Submitted filename: Response to Reviewers.docx [file pone.0243435.s005.docx]

We would like to thank the reviewers for their thoughtful and insightful comments on this study. We have made extensive revisions and agree with the reviewers’ points that these changes would strengthen the paper. The reviewers’ **comments are below in black** and our **responses and specific actions are noted in blue**.

-------------------------------------------------------------------------------------------------------------------------------

Reviewer #1: There is very limited literature on Embedded Support Features, which can provide critical supports to struggling readers for understanding new content and participating in learning activities. Therefore, this study, which explores the efficacy of a tablet-based text annotation reading tool in intervention research, to help children decode novel words, fills a gap in the literature on embedded digital supports. The study makes a convincing case that while the edtech market has grown tremendously as new apps are being released every year and federal funds are being devoted to provide infrastructure for even greater involvement with digital media in schools, many of the edtech tools “lacked scientific backing”.

While the study rightfully argues that edtech tools should go beyond just being entertaining and should be backed by research-based best practices, it fails to provide some literature on the best-practices. The current study lacks important and needed detail regarding how the existing literature would be evaluated to arrive at meaningful conclusions. For example, the study should refer to the extensive scientific literature on what works for struggling readers in terms of best practices as well as existing studies on embedded digital support tools. A review of the existing literature on digital supports would have provided a better understanding of what is known--which digital features or supports have been found to have promise to support struggling students’ learning and under what conditions. The study made some references to such tools/studies in the Discussion section, which should have been presented in the Literature Review section.

**Thank you for this critique - We have revised the Introduction to not only provide aspects that were previously left for the Discussion but have also provided a more comprehensive literature review on embedded supports. For example, we now write:**

**“As a means of scaffolding learning in the classroom, technological tools provide limitless practice [19–21] that can be individualized [17,22,23] to optimize for an individual reader. Additionally, modern computational tools utilizing speech recognition and synthesis application programming interfaces (APIs), allow for embedded tools to be provided in real-time for any given text. One promising avenue for technology has been the use of embedded support features such as visual images to facilitate learning sound-symbol correspondence. For examples, *Trainertext*, a program in the United Kingdom which provides visual mnemonics above each phoneme in a given text, saw significant improvement after 10 months of exposure and at-home practice [24]. By providing a visual scaffold, the authors concluded that readers could leverage a phonics-based text adaptation to improve their decoding skill. Moreover, *SeeWord Reading app*, a digital tool which uses a picture-embedded font to demonstrate grapheme-phoneme correspondence [25], has demonstrated efficacy in studies performed both in Singapore and the United States [26]. Both *Trainertext* and *SeeWord Reading*, demonstrate the utility of an embedded support to provide concrete visual relationships between written text and spoken language: a foundational skill for literacy. Together these studies demonstrate that technologies focusing at the level of the phoneme and the syllable (or rime) not only benefit reading performance, but also adhere to known learning and pedagogical principles [26,27].” [p5, 3-19]**

I agree with the authors who argue that “there is great need for researchers studying literacy development and reading disabilities to work with tech developers on the design of tools that are grounded in the extensive scientific on what works for struggling readers, systematically test their effectiveness, and contribute to the development of standards of practice for educational apps targeted at literacy”. However, even though we have “evidence-based practices (e.g., phonemic awareness, phonics)”, we cannot assume that the best practices identified in non-digital contexts may easily apply or be expanded within digital learning contexts. The study makes such an assumption.

**This is an important point to address - although we do agree that not all non-digital pedagogy can be applied to digital contexts, we believe that to be at the heart of the need for systematic testing and the establishment of standards of practice.**

**The sentence you reference is now expanded to include the following:**

**“Thus, there is great need for researchers studying literacy development and reading disabilities to work with tech developers on the design of tools that are grounded in the extensive scientific literature on what works for struggling readers, systematically test their effectiveness, and contribute to the development of standards of practice for educational apps targeted at literacy. Recent metanalyses demonstrate much promise for digital solutions in the context of literacy, yet also describe the multitude of ways that technology is an inappropriate substitute for many aspects of pedagogy [9,16,17]. In parallel with research outside of technology, the onus for researchers is to rigorously test digital solutions to discover “what works best in which programs for what students under which conditions” [18].” [p4,16-p5,2]**

The study states that “Intervention participants completed an extended training period” but it is not clear what “extended” means.

**Because the control group was not instructed in the use of the digital tool their training period was limited to instruction in how to read the at-home practice passages. The extended, or longer, training session for the intervention group is expanded upon in the “Training program” section that follows. In short, ‘extended’ is just used as a contrast to ‘brief’ used earlier in the paragraph. We have taken the following action to clarify this in the manuscript:**

**“Control participants completed a brief training in use of the tablet then a two-week, at-home practice program without the Sound It Out tool. Intervention participants completed the full training period that included both the use of the tablet as well as a formalized introduction to and practice with the Sound It Out cue prior to an identical at-home practice program with the tool turned on.”[p 11, 4-8]**

One of the weaknesses of the study is to conclude that 100 minutes of exposure over the two-week practice period is enough to make conclusions about the efficacy of a tablet-based text annotation reading tool. The result could simply be due to the novelty effects, which pose a threat to external validity because they make it difficult to know if the results of the study are due to a treatment that works or due to the novelty of a treatment.

**This is a valid point and, in response, have revised our discussion of the limitations of the exposure period to say:**

**“Thus, although the training and practice periods were enough to encourage effective use, they were not enough to ensure automatic and fluent use in a natural setting, and by extension, were insufficient to make general claims on efficacy of Sound it Out for supporting long-term growth in reading skills.” [p 22,22-p23,2]**

Reviewer #2: The authors describe a small-scale randomized control trial with 40 struggling readers aged 8-12 who are split across two intervention groups. The aim was to investigate the effect of annotating text with phonemic cues in a tablet app by allowing children to play for a two-week intervention phase. Their results indicate that there is a benefit on untimed pseudoword decoding accuracy, but no effects on word decoding accuracy or connected text reading accuracy and speed.

The manuscript is well written, the experimental design was preregistered and all data was made available.

**Thank you for the positive feedback.**

There are some shortcomings in this study, some of which relate to the experimental setup itself, and others to unjustified deviations from the study preregistration.

Major:

1) The manuscript itself does not contain any research questions and hypothesis and only refers to the preregistration on OSF. This should be changed and implemented into the manuscript. Furthermore, the preregistration contains an additional question relating to individual phonological skills which is not touched upon in the manuscript.

**We appreciate this feedback and have added content to address this short coming in the introduction and throughout the paper. The additions include the following:**

**“As reflected in the preregistered report, the study aimed to answer the following questions: Can struggling readers use phonemic cues to improve reading fluency?, Do struggling readers benefit from phonemic cues when decoding difficult words without time constraints?, and Can we predict which individuals will benefit from the tool based on a standardized battery of reading-related assessments? We hypothesized that the phonemic cue would aid struggling readers in more accurately reading short passages, and with repeated reading, will increase their reading rate. Further, we hypothesized that the phonemic cue would aid struggling readers in more accurately decoding individual real and pseudo-words and that this benefit will relate to the amount of practice they have had with the tool. Finally, regarding individual differences, we hypothesized that those struggling readers that have a specific impairment in phonological processing would benefit the most from the support provided by this tool.” [p6, 13-23]**

**The following sections have been added to the results to address the previously missing third research question:**

**“Regarding our final question on subject characteristics that predict individual response, a correlation analysis revealed no significant relationships between our baseline predictors (age, WASI-II Full Scale-2, and CTOPP-2) and improvement in real-word decoding. For pseudo-word decoding, there were negative correlations with the CTOPP-2 Phonological Awareness (PA) (r = -0.52, p = 0.018) and Phonological Memory (PM) (r = -0.48, p = 0.034) composite measures as well as the WASI-II Full Scale – 2 score (r = -0.49, p = 0.027), but these effects were not significant after correcting for multiple comparisons.” [p17, 13-19]**

**“Moreover, prediction analyses suggest that this effect is more pronounced for those participants with more pronounced impairments in phonological processing and lower IQ (though these effects did not surpass our adjusted significance threshold of p < 0.0125).“ [p17,23-p18,3]**

**“Correlation analyses revealed only a near-significant negative relationship between age and word reading accuracy (r = -0.55, p = 0.035) suggesting that younger children may benefit more from Sound it Out.” [p19, 3-5]**

2) As far as I understand from the manuscript the word lists and reading passages at post-training assessment were carried out with the phonemic cues present as depicted in Figure 2. It remains unclear whether this feature was only activated for the intervention group (1) or both groups (2). For 1) this would mean that what is being measured is the effect of cueing but not the generalization of the training on un-cued reading. Option 2) would mean that control children are confronted with this annotation for the first time at an assessment moment, which would increase their cognitive load and put them at a disadvantage. In either case, the impact of training should be assessed without cueing in a real-world reading scenario.

**We apologize that this aspect was not made more clear. We have amended the paragraph on page 10 detailing the study design to instead say:**

**“After two weeks, all participants returned for a post-intervention visit where the intervention participants were tested with the cue, while control participants experienced the normal text condition for all study stimuli. As the goal of this study was to test proof-of-concept for a digitally-embedded phonetic cue in a small scale RCT, generalization to un-cued reading in the intervention group was not tested.” [p11, 8-12]**

**Examining how reading practice with the benefit of phonemic cues generalizes to reading without the cues is an important future direction. The best way to achieve this would be a long-term longitudinal study where children are taught how to use the tool, provided the tool to use over the course of a school year, and then their reading skills are tested at the end of the year. This long-term longitudinal design would be the critical test because we would not expect generalization after such a short time period. One thing that the dyslexia intervention literature makes clear is that major gains cannot be achieved with short-term interventions. This goes beyond the scope of the present work as the goals here were to (a) develop the tool and (b) validate that children could leverage it to read words that they otherwise couldn’t read. Though we have reason to be optimistic that long-term practice would generalize: A similar study by Messer and Nash described generalization after 10 months of supervised practice and exposure. We would hope to observe similar effects with similar exposure to Sound it Out.**

3) P13:L21: "a threshold of 0.025 was defined in the preregistered report to adjust for multiple comparisons". The report contains a threshold of 0.0125 which would render the sole significant finding not significant. Where does this deviation come from?

**This was an unfortunate oversight and we thank you for drawing our attention to this discrepancy. The threshold in the preregistration is correct and has been amended throughout the paper, with conclusions/interpretations adjusted to reflect the findings did not meet our significance threshold.**

**We thank the reviewer for catching the minor deviations from our pre-registration. Upon a careful re-read we realized that over the months between writing the registration and finalizing all of the analysis code we made some small deviations to (a) our model specifications, (b) our statistical thresholds and (c) the statistical approach to the prediction analysis. In the revision we now stick precisely to everything laid out in the pre-registration. As it happens, the pseudo-word result is significant at p=0.003. We have also added transparency to the statistics section to reflect the changes we made to the analysis plan as well as a “Transparent Changes” document to our public OSF project repository.**

Minor:

Introduction:

Adding phonemic cues in form of symbols above text is not necessarily bound to digital media per se and could equally be done on paper. It remains unclear why there is a focus on implementing this inside an app. How does this relate to previous literacy intervention work? Has this been done on paper and were there comparable findings?

**In response to this feedback (as well as the other reviewer’s) we have amended the Introduction to have a broader literature review on embedded text supports. This expansion details that this area has been successful in both domains and adds further explanation of digital means in their ability to utilize speech APIs to generate embedded supports in real-time for limitless text input.**

**“As a means of scaffolding learning in the classroom, technological tools provide limitless practice [19–21] that can be individualized [17,22,23] to optimize for an individual reader. Additionally, modern computational tools utilizing speech recognition and synthesis application programming interfaces (APIs), allow for embedded tools to be provided in real-time for any given text. One promising avenue for technology has been the use of embedded support features such as visual images to facilitate learning sound-symbol correspondence. For examples, *Trainertext*, a program in the United Kingdom which provides visual mnemonics above each phoneme in a given text, saw significant improvement after 10 months of exposure and at-home practice [24]. By providing a visual scaffold, the authors concluded that readers could leverage a phonics-based text adaptation to improve their decoding skill. Moreover, *SeeWord Reading app*, a digital tool which uses a picture-embedded font to demonstrate grapheme-phoneme correspondence [25], has demonstrated efficacy in studies performed both in Singapore and the United States [26]. Both *Trainertext* and *SeeWord Reading*, demonstrate the utility of an embedded support to provide concrete visual relationships between written text and spoken language: a foundational skill for literacy. Together these studies demonstrate that technologies focusing at the level of the phoneme and the syllable (or rime) not only benefit reading performance, but also adhere to known learning and pedagogical principles [26,27].” [p5, 3-19]**

Method:

P6:L17: The preregistration report mentions 1.5SD below the mean as an inclusion criterion for being a struggling reader, here it is 1SD. Where does this deviation come from?

**This deviation (with clarification added to page 8 of the manuscript) was due to issues of subject recruitment as well as a desire to better include the heterogeneity of struggling readers in our subject population. The Participant section of the methods now reads as follows:**

**“A threshold of 1 SD, rather than the 1.5 SD threshold defined in our preregistration, was adopted in order to better account for the heterogeneity in the struggling reading population and to expedite participant recruitment.” [p8,2-4]**

The group characteristics provided in Table 1 do not contain p-values to show that the groups do not differ from one another. Could you also explain the randomization/matching used for group assignment? Were there any constraints so that the groups would end up being comparable?

**We have amended the table note to clarify that t-tests were performed on the characteristics and that no significant difference was found. Further, we have amended the Study Design section of the methods to describe that randomization was unconstrained except for a rule for sibling participants to be assigned to the same group to ensure adherence.**

In the discussion you mention that many participants were receiving supplemental instruction for their reading difficulties. Can you specify how many and how their distribution among the two groups were? Potential gains may ultimately also stem from simultaneous speech therapy if the groups are not balanced.

**Although we mention that many students had received supplemental instruction, this point was primarily to describe why the very short timeframe of 2-weeks was insufficient to produce more substantive effects, and to explain the impact of a novel strategy in a population that tends to have heightened exposure to reading curricula. We do agree that in a larger scale study that is aiming to test the efficacy of a tool it would be important to collect detailed data on any supplemental instruction received, but given the nature of this study we do not believe a more thorough analysis is warranted. We do, however, know that none of the participants were receiving speech therapy.**

**We have revised the manuscript to add more clarity:**

**“Many participants in the study have received supplemental instruction previously and have learned strategies for approaching new words. As a novel strategy, the phonemic cue may have been overridden when children were asked to read continuously and for comprehension.” [p23,5-8]**

Given that your sample is quite heterogenous with an age range of 8-12 years and the intention to include a broad range of struggling readers it might be worthwhile to add additional co-variates to your models to support a more causal and generalizable interpretation of your intervention (e.g. for age or pre-test phonological awareness skills - which was also an unmentioned research question).

**We performed the recommended analyses and although we found that a model including age and initial phonological awareness was a better fit to the data for some outcome measures, the statistics remain unchanged using either model.**

Could you provide additional information on the custom word lists you created? E.g. frequency information for the words and neighborhood density for the pseudowords. Furthermore, reliability information would also be relevant, i.e. Cronbach's alpha, split-half reliability or correlation between lists.

**As requested, tables with frequency information has been added to the Supplement. Moreover, reliability information is added to the Methods section on page 14 to reflect high inter-list correlations for both real and pseudo word lists.**

**“Post-hoc analysis revealed that performance was highly reliable across the different word lists: performance was highly correlated for the two lists of real words (r = 0.86, p = 5.73*10^-24^) and pseudo words (r = 0.79, p = 3.645*10^-18^) in each visit.” [p14, 4-6]**

Matlab script: Is there a specific reason why the models where fit with method 'ML'? This is only useful for stepwise model comparison and final models for publication should be fitted with REML as this gives a less biased estimation and more robustness towards outliers.

**Thank you for this feedback. Although analysis of model comparisons was performed with ML, we should have performed the resulting analysis should have been done in REML. We have amended the statistics to reflect analyses fit with REML.**

Results:

You mention missing data. Could you specify which variables from how many subjects are affected by this?

**Data were missing for 12 passages from seven participants (word accuracy and rate information) due to testing fatigue or inability to complete the passage.**

I much appreciate access to the raw data and code! It would be nice if you added a code book indicating what measure each variable contains.

**Of course! We have added a code book to the public GitHub repository.**

P14:L3: Looking at the numbers of played stories (mean=13/12, SD=6) it seems that intervention fidelity was not very high. Could you provide more information on that? How many minutes of training would this translate to? What was the range of minimum and maximum exposure? Did children adhered to the instruction of playing one story per day? What happened to children that played less? Did they start playing each day and then stop after a few days? Were there children that did not play for a week and then played 10 session on the last day?

**This was a major challenge in our study as we had planned to use the tracking data from Amazon but found it highly unreliable in measuring usage statistics due the tool being accessed within the browser. Fortunately, we had decided to collect comprehension data which ended up being a great method of corroborating the tracking data that was able to indicate the story being open on the browser. You’re correct that fidelity to the cadence of reading was poor with most participants ‘catching up’ in the second week, but most participants did complete the necessary 10 stories that were required of them within the 2-week period (34/40). Unfortunately, due to the unreliability of the tracking data we aren’t able to say how many minutes of exposure they had; only that the number of passages completed with understanding (per the comprehension quizzes).**

P14:L3: Instead of looking into correlations I would suggest including app exposure as a covariate into your models.

**This would be ideal, but given the issues above, the correlation analysis was the best approach given the data we had access to.**

P14:L10: Thank you for providing an effect size. It would be informative if you could also provide these for all other discussed effects.

**Effect sizes for real word decoding, word reading accuracy, and word reading rate are now listed in the manuscript.**

**“Although the effect sizes were moderate (Cohen’s d = 0.74 for pseudoword decoding, d = 0.57 for real word decoding), …” [p18, 3-5]**

**“Lack of significance is reflected in small effect size of d = 0.36 for accuracy and 0.26 for rate.” [p19,1-2]**

The preregistration report states: "Based on pilot data it is reasonable to expect that the intervention will help subjects read 5-10 additional words on the untimed word lists." as well as "subjects differ, on average, by +/- 3 words on repeated administrations of the word lists". Based on these numbers a statistical power of 0.9 was estimated.

In the actual study the results are much lower: 1 additional word and 3 additional pseudowords, which falls into the range of the expected test-retest reliability you mentioned. Which makes me wonder how robust is this effect? The actual power will be much lower and the reported effect size of d=0.74 seems way too large for such a short intervention period. I would be very careful at overinterpreting these findings as they are probably not generalizable.

**This is an important aspect of our findings and we are very careful to describe how these findings suggest proof-of-concept for the embedded annotation, did not reveal generalization, and do not demonstrate general efficacy.**

Discussion:

Given the comments above, possible effects should be discussed with more caution. It also remains unclear what was actually measured with the experimental design (see major point 2 above). This should be made clearer when discussing potential effects.

**This is valuable advice - as such the discussion (including conclusions and interpretation) have been amended to apply more caution to our findings.**

**“However, these results were not significant (after multiple comparison correction) and, therefore, are insufficient to support any conclusions regarding the relationship between subject characteristics and benefits conferred by phonemic cues. Together, although most analyses failed to meet our adjusted significance threshold, data suggests that participants were able to effectively use the cues in isolated situations, but the tool did not become sufficiently automatic to produce significant gains in passage reading fluency.” [p19,22-p20,4]**

**“Albeit promising, these results should be interpreted cautiously: Our power analysis indicated that we only had sufficient power to detect relatively large effects and many of the analyses were likely underpowered. Thus, future studies are needed with larger sample sizes to provide more conclusive results. Moreover, two additional points merit further investigation. First, future studies should more efficiently monitor practice adherence and cadence at home to better explore the relationship between exposure and reading-related measures. Second, given the short intervention period, we did not examine generalization to reading improvements without the cue and across different aspects of skilled reading. We only investigated whether the cue could be effectively used to decode more complex words. Thus, examining long-term learning effects and generalization to a variety of different contexts is an important future direction.” [p22, 9-19]**

**“We had anticipated that the cue would require limited exposure, but our results are in stark contrast with previous studies that have instituted a more comprehensive, extended training program and observed significant benefit to fluent reading [24,50]. Thus, although the training and practice periods were enough to encourage effective use, they were not enough to ensure automatic and fluent use in a natural setting, and by extension, were insufficient to make general claims on efficacy of Sound it Out for supporting long-term growth in reading skills.” [p22,20-p23,2]**

**“In aggregate, these findings represent proof-of-concept for this novel approach to assisting struggling readers by merging the extensive evidence base on effective literacy instruction and the affordances that technology lends to the educational arena. Not only did it prove promising in improving decoding performance with very limited practice, but it also was observed to be non-detrimental to passage reading, meaning that it was not too cognitively demanding. Future research should focus on optimizing training and practice to produce gains that will extend beyond isolated single word decoding and lead to more confident, fluent readers.“ [p24,3-10]**

The lack of research questions in the manuscript makes the discussion rather unspecific. Here you should get back to your initial questions and hypothesis.

**The first paragraph of the discussion has been amended to mirror the research questions introduced in the introduction.**

P15:L11: "In connected text reading, growth was observed for all participants but without a significant difference between groups." These effects are essentially zero (0.01 - 0.04 words per minute extra), so they don’t have a practical relevance.

**The sentence has been amended to instead say:**

**“In connected text reading, stable performance was observed for all participants with no significant difference between groups.” [p19,16-17]**

P16:L16: There are many different effect size measures which differ in their scales. Please mention which one is meant.

**The effect size has been labelled with the appropriate qualifier as being Cohen’s d.**

I'm missing a limitations section where you discuss possible lack of power, adherence to daily app use and the need for replication in a bigger sample.

**The existing limitations section has been revised to include the following:**

**“Albeit promising, these results should be interpreted cautiously: Our power analysis indicated that we only had sufficient power to detect relatively large effects and many of the analyses were likely underpowered. Thus, future studies are needed with larger sample sizes to provide more conclusive results. Moreover, two additional points merit further investigation. First, future studies should more efficiently monitor practice adherence and cadence at home to better explore the relationship between exposure and reading-related measures. Second, given the short intervention period, we did not examine generalization to reading improvements without the cue and across different aspects of skilled reading. We only investigated whether the cue could be effectively used to decode more complex words. Thus, examining long-term learning effects and generalization to a variety of different contexts is an important future direction.” [p22, 9-19]**
